# Supplementary material for: Reduced progranulin increases tau and α-synuclein inclusions and alters mouse tauopathy phenotypes via glucocerebrosidase
Source: Nat Commun. 2024 Feb 16;15:1434. doi: 10.1038/s41467-024-45692-3 (PMC10873339; doi:10.1038/s41467-024-45692-3)
Supplement: Supplementary file 1 — Supplementary Information [file 41467_2024_45692_MOESM1_ESM.pdf]

Supplementary Information

Reduced progranulin increases tau and α-synuclein inclusions and alters mouse tauopathy phenotypes via glucocerebrosidase

Hideyuki Takahashi[1], Sanaea Bhagwagar[1,2], Sarah H. Nies[1,3], Hongping Ye[4], Xianlin Han[4,5], Marius T. Chiasseu[1], Guilin Wang[6], Ian R. Mackenzie[7], and Stephen M. Strittmatter[1,*]

Supplementary Figures 1-21

Supplementary Tables 1-2

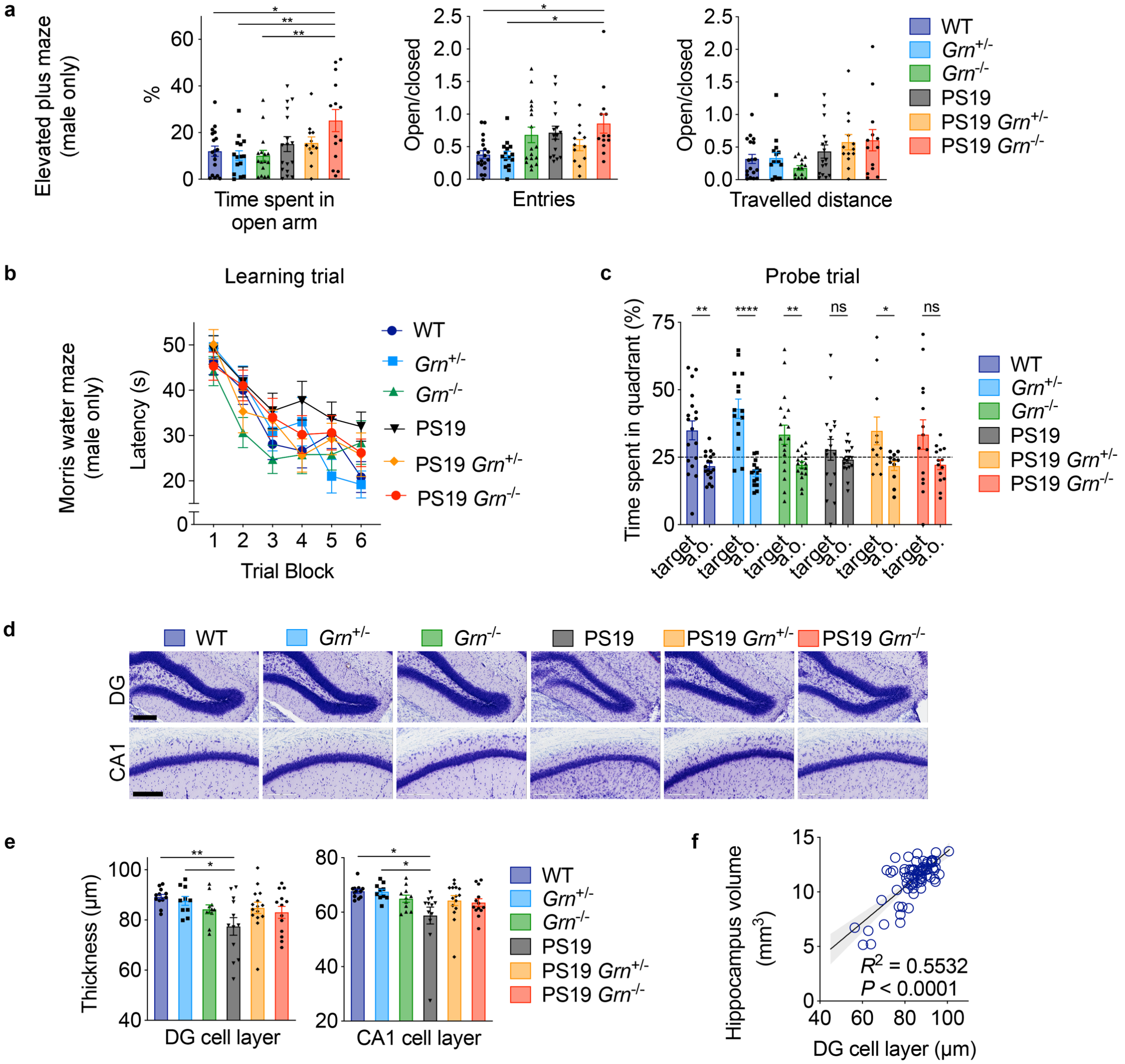

Supplementary Fig. 1: PGRN reduction exacerbates disinhibition while improving a memory deficit in male PS19 mice

a. Elevated plus maze (EPM) of male 6 genotypes (WT, *Grn*$^{+/-}$, *Grn*$^{-/-}$, PS19, PS19 *Grn*$^{+/-}$, and PS19 *Grn*$^{-/-}$ mice) at 10-11 months of age showing time spent in the open arms (%), and ratio of number of entries into the open versus closed arms ration of distance traveled in the open versus closed arms of the maze. Mean ± SEM, n = 18 mice (WT), n = 15 mice (*Grn*$^{+/-}$), n = 18 mice (*Grn*$^{-/-}$), n = 18 mice (PS19), n = 13 mice (PS19 *Grn*$^{+/-}$), and n = 14 mice (PS19 *Grn*$^{-/-}$), *p =0.0225 (time spent in open arms, WT vs. PS19 *Grn*$^{-/-}$), **p = 0.0088 (time spent in open arms, *Grn*$^{+/-}$ vs. PS19 *Grn*$^{-/-}$), **p = 0.0072 (time spent in open arms, *Grn*$^{-/-}$ vs. PS19 *Grn*$^{-/-}$), *p =0.0118 (entries to open arms, WT vs. PS19 *Grn*$^{-/-}$), *p = 0.0156 (entries to open arms, *Grn*$^{+/-}$ vs. PS19 *Grn*$^{-/-}$); One-way ANOVA with Tukey's post hoc test.

b. Morris water maze (MWM) learning trial of male 6 genotypes at 10-11 months of age. Spatial learning is plotted as latency to find hidden platform. Mean ± SEM, n = 18 mice

(WT), n = 15 mice (*Grn*$^{+/-}$), n = 18 mice (*Grn*$^{-/-}$), n = 17 mice (PS19), n = 11 mice (PS19 *Grn*$^{+/-}$), and n = 14 mice (PS19 *Grn*$^{-/-}$).

c. MWM probe trial of male 6 genotypes at 10-11 months of age. Percentage of time spent in the target quadrant and averaged time spent in all other (a.o.) quadrants. Mean ± SEM, n = 18 mice (WT), n = 15 mice (*Grn*$^{+/-}$), n = 18 mice (*Grn*$^{-/-}$), n = 17 mice (PS19), n = 11 mice (PS19 *Grn*$^{+/-}$), and n = 14 mice (PS19 *Grn*$^{-/-}$), **p = 0.0018 (WT), ****p = 0.000007 (*Grn*$^{+/-}$), **p = 0.0075 (*Grn*$^{-/-}$), *p = 0.0330 (PS19 *Grn*$^{+/-}$); Two-tailed unpaired t test with Welch's correction.

d. Representative images of Nissl staining of the dentate gyrus (DG) and CA1 region of hippocampus from 6 genotypes. Bar, 200 μm.

e. Thickness of the DG granule cell layer and CA1 pyramidal neuronal layer in 6 genotypes at 9-11 months of age. Mean ± SEM, n = 12 mice (WT), n = 10 mice (*Grn*$^{+/-}$), n = 11 mice (*Grn*$^{-/-}$), n = 12 mice (PS19), n = 15 mice (PS19 *Grn*$^{+/-}$), and n = 13 mice (PS19 *Grn*$^{-/-}$), **p = 0.0083 (DG, WT vs. PS19), *p = 0.0486 (DG, *Grn*$^{+/-}$ vs. PS19), *p = 0.0102 (CA1, WT vs. PS19), *p = 0.0200 (CA1, *Grn*$^{+/-}$ vs. PS19); One-way ANOVA with Tukey's post hoc test.

f. Correlation between DG granule cell layer thickness and hippocampal volume. n = 73 mice, R^2 = 0.5532, p < 0.0001; Pearson correlation analysis.

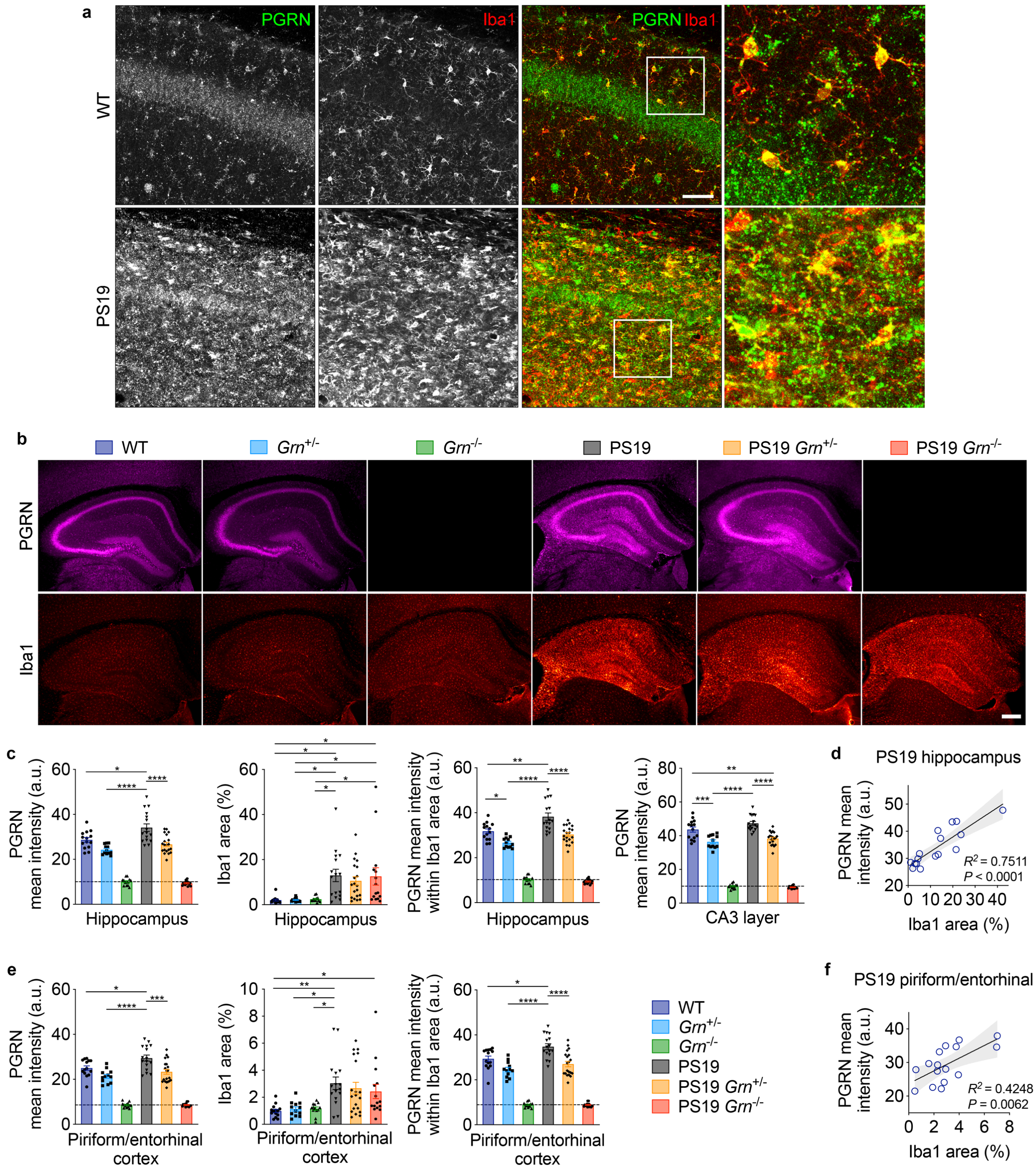

Supplementary Fig. 2: PGRN is increased in PS19 mice mainly due to microgliosis and is decreased in PS19 *Grn*$^{+/-}$ mice

a. Representative confocal images of PGRN and Iba1 co-staining of the C1A region of hippocampus of 11-month-old WT and PS19 mice. Bar, 50 μm.

b. Representative images of PGRN and Iba1 co-staining of the hippocampus of 6 genotypes (WT, *Grn*$^{+/-}$, *Grn*$^{-/-}$, PS19, PS19 *Grn*$^{+/-}$, and PS19 *Grn*$^{-/-}$ mice) at 9-12 months of age. Bar, 300 μm.

c. Quantification of PGRN mean intensity, Iba1 area (%), and PGRN mean intensity within Iba1 area PGRN mean intensity of CA2 and CA3 neuronal layer in the hippocampus of 9-12-month-old 6 genotypes. Mean ± SEM, n = 14 mice (WT), n = 12 mice (*Grn*$^{+/-}$), n = 13 mice (*Grn*$^{-/-}$), n = 16 mice (PS19), n = 20 mice (PS19 *Grn*$^{+/-}$), and n = 15 mice (PS19 *Grn*$^{-/-}$), *p = 0.0122 (PGRN mean intensity, WT vs. PS19), ****p = 0.000005 (PGRN mean intensity, *Grn*$^{+/-}$ vs. PS19), ****p = 0.000084 (PGRN mean intensity, PS19 vs. PS19 *Grn*$^{+/-}$), *p = 0.0110 (Iba1 area, WT vs. PS19), *p = 0.0191 (Iba1 area, WT vs. PS19 *Grn*$^{-/-}$), *p = 0.0175 (Iba1 area, *Grn*$^{+/-}$ vs. PS19), *p = 0.0286 (Iba1 area, *Grn*$^{+/-}$ vs. PS19 *Grn*$^{-/-}$), *p = 0.0198 (Iba1 area, *Grn*$^{-/-}$ vs. PS19), *p = 0.0326 (Iba1 area, *Grn*$^{-/-}$ vs. PS19 *Grn*$^{-/-}$), *p = 0.0277 (PGRN mean intensity within Iba1 area, WT vs. *Grn*$^{+/-}$), **p = 0.0012 (PGRN mean intensity within Iba1 area, WT vs. PS19), ****p = 0.00000005 (PGRN mean intensity within Iba1 area, *Grn*$^{+/-}$ vs. PS19), ****p = 0.00001 (PGRN mean intensity within Iba1 area, PS19 vs. PS19 *Grn*$^{+/-}$), ***p = 0.0008 (PGRN mean intensity within CA3 cell layer, WT vs. *Grn*$^{+/-}$), **p = 0.0064 (PGRN mean intensity within CA3 cell layer, WT vs. PS19 *Grn*$^{+/-}$), ***p = 0.0000003 (PGRN mean intensity within CA3 cell layer, *Grn*$^{+/-}$ vs. PS19), ****p = 0.00001 (PGRN intensity within CA3 cell layer, PS19 vs. PS19 *Grn*$^{+/-}$); One-way ANOVA with Tukey's post hoc test. For PGRN intensity measurements, 2 genotypes *Grn*$^{-/-}$ and PS19 *Grn*$^{-/-}$ were removed from statistical tests.

d. Correlation between Iba1 area and PGRN mean intensity in the hippocampus of PS19 mice. n = 16 mice, R^2 = 0.7511, p = 0.00001; Two-tailed Pearson correlation analysis.

e. Quantification of PGRN mean intensity, Iba1 area (%), PGRN mean intensity within Iba1 area in the piriform/entorhinal cortex of 9-12-month-old 6 genotypes. Mean ± SEM, n = 14 mice (WT), n = 12 mice (*Grn*$^{+/-}$), n = 13 mice (*Grn*$^{-/-}$), n = 16 mice (PS19), n = 20 mice (PS19 *Grn*$^{+/-}$), and n = 15 mice (PS19 *Grn*$^{-/-}$), *p = 0.0435 (PGRN mean intensity, WT vs. PS19), ****p = 0.00008 (PGRN mean intensity, *Grn*$^{+/-}$ vs. PS19), ***p = 0.0008 (PGRN mean intensity, PS19 vs. PS19 *Grn*$^{+/-}$), **p = 0.0046 (Iba1 area, WT vs. PS19), *p = 0.0217 (Iba1 area, WT vs. PS19 *Grn*$^{+/-}$), *p = 0.0173 (Iba1 area, *Grn*$^{+/-}$ vs. PS19), *p = 0.0154 (Iba1 area, *Grn*$^{-/-}$ vs. PS19), *p = 0.0190 (PGRN mean intensity within Iba1 area, WT vs. PS19), ****p = 0.000006 (PGRN mean intensity within Iba1 area, *Grn*$^{+/-}$ vs. PS19), ****p = 0.00008 (PGRN mean intensity within Iba1 area, PS19 vs. PS19 *Grn*$^{+/-}$); One-way ANOVA with Tukey's post hoc test. For PGRN intensity measurements, 2 genotypes *Grn*$^{-/-}$ and PS19 *Grn*$^{-/-}$ were removed from statistical tests.

f. Correlation between Iba1 area and PGRN mean intensity in the piriform/entorhinal cortex of PS19 mice. n = 16 mice, R^2 = 0.4248, p = 0.0062; Two-tailed Pearson correlation analysis.

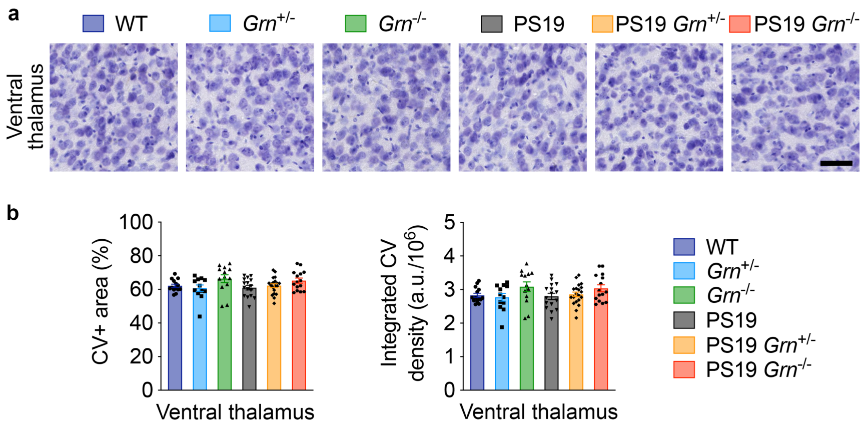

Supplementary Fig. 3: Thalamic cell density is not affected by P301S tau and/or PGRN deficiency

a. Representative Nissl staining of the ventral thalamic region of 6 genotypes (WT, *Grn*$^{+/-}$, *Grn*$^{-/-}$, PS19, PS19 *Grn*$^{+/-}$, and PS19 *Grn*$^{-/-}$ mice). Bar, 50 µm.

b. Cresyl violet (CV)-positive area or integrated CV density of 6 genotypes at 9-12 months of age. Mean ± SEM, n = 14 mice (WT), n = 12 mice (*Grn*$^{+/-}$), n = 13 mice (*Grn*$^{-/-}$), n = 16 mice (PS19), n = 20 mice (PS19 *Grn*$^{+/-}$), and n = 15 mice (PS19 *Grn*$^{-/-}$). Six groups were not significantly different by one-way ANOVA.

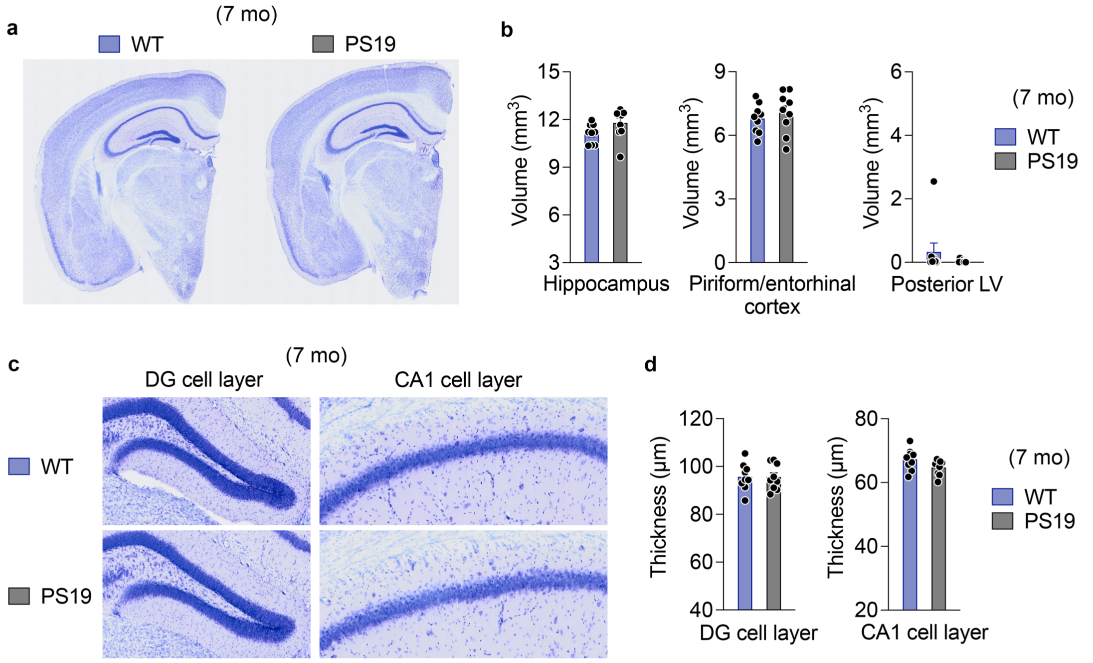

Supplementary Fig. 4: No significant neurodegeneration in 7-month-old PS19 mice

a. Representative Nissl staining of brain sections from 7-month-old 6 WT and PS19 mice.

b. Volumes of the hippocampus, piriform/entorhinal cortex, and posterior lateral ventricle (LV) in 7-month-old WT and PS19 mice. Mean ± SEM, n = 9 mice per genotype. Two genotypes were not significantly different by two-tailed unpaired t-test.

c. Representative images of Nissl staining of the dentate gyrus (DG) and CA1 region of hippocampus in 7-month-old WT and PS19 mice.

d. Thickness of the DG granule cell layer and CA1 pyramidal neuronal layer in 7-month-old WT and PS19 mice. Mean ± SEM, n = 9 mice per genotype. Two genotypes were not significantly different by two-tailed unpaired t-test.

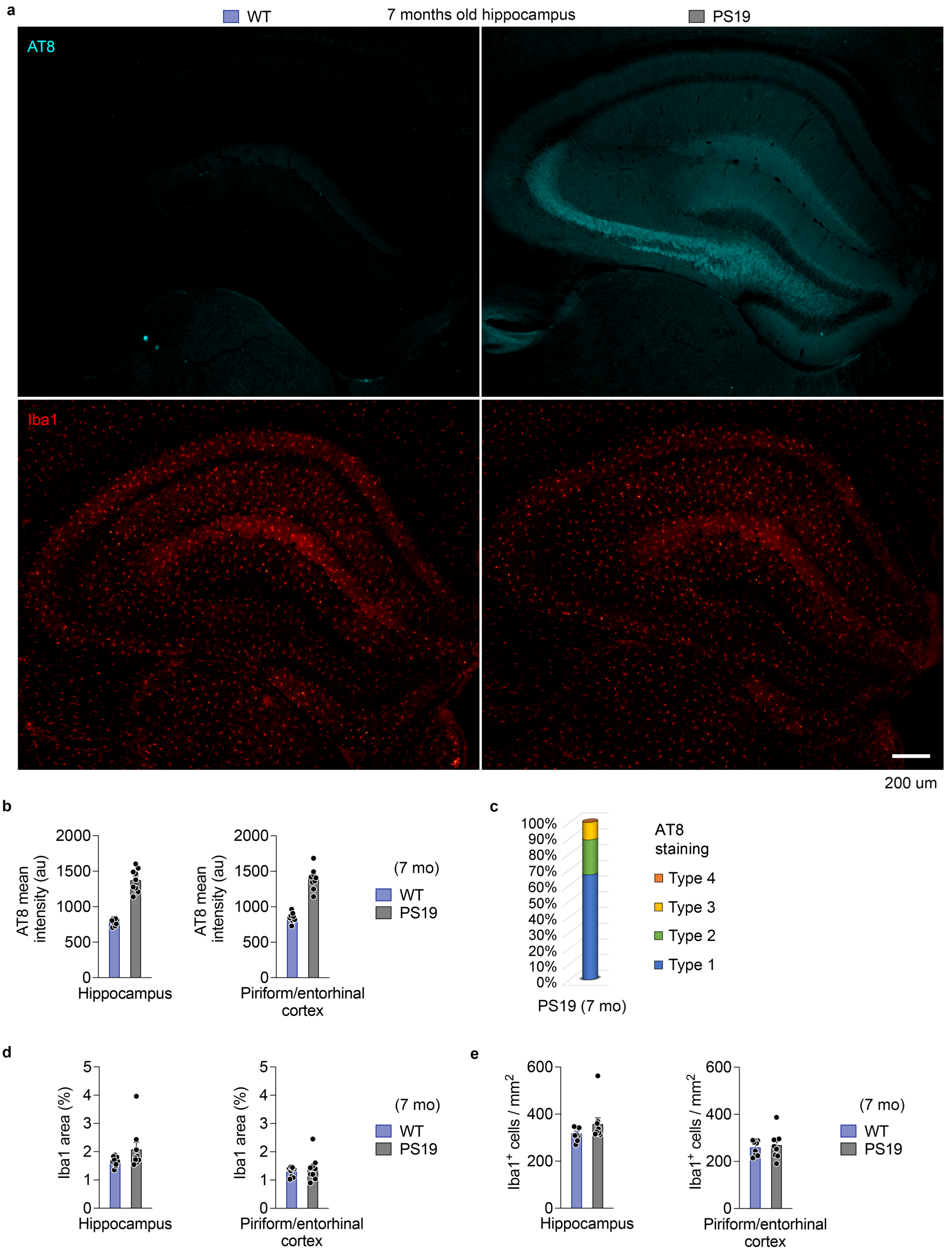

Supplementary Fig. 5: Tau pathology and microgliosis in 7-month-old PS19 mice

- **a.** Representative images of AT8 and Iba1 co-staining in the hippocampus of WT and PS19 mice at 7 months of age. Bar, 200 μm.
- **b.** AT8 mean intensity in the hippocampus and piriform/entorhinal cortex of WT and PS19 mice at 7 months of age. Mean ± SEM, n = 9 mice per genotype.
- **c.** Distribution of the AT8 staining types in the hippocampus of PS19 mice at 7 months of age. n = 9 mice.
- **d.** Quantification of Iba1-positive area in the hippocampus and piriform/entorhinal cortex of 7-month-old WT and PS19 mice. Mean ± SEM, n = 9 mice per genotype. Two genotypes were not significantly different by two-tailed unpaired t-test.
- **e.** Quantification of the number of Iba1-positive microglia in the hippocampus and piriform/entorhinal cortex of 7-month-old WT and PS19 mice. Mean ± SEM, n = 9 mice per genotype. Two genotypes were not significantly different by two-tailed unpaired t-test.

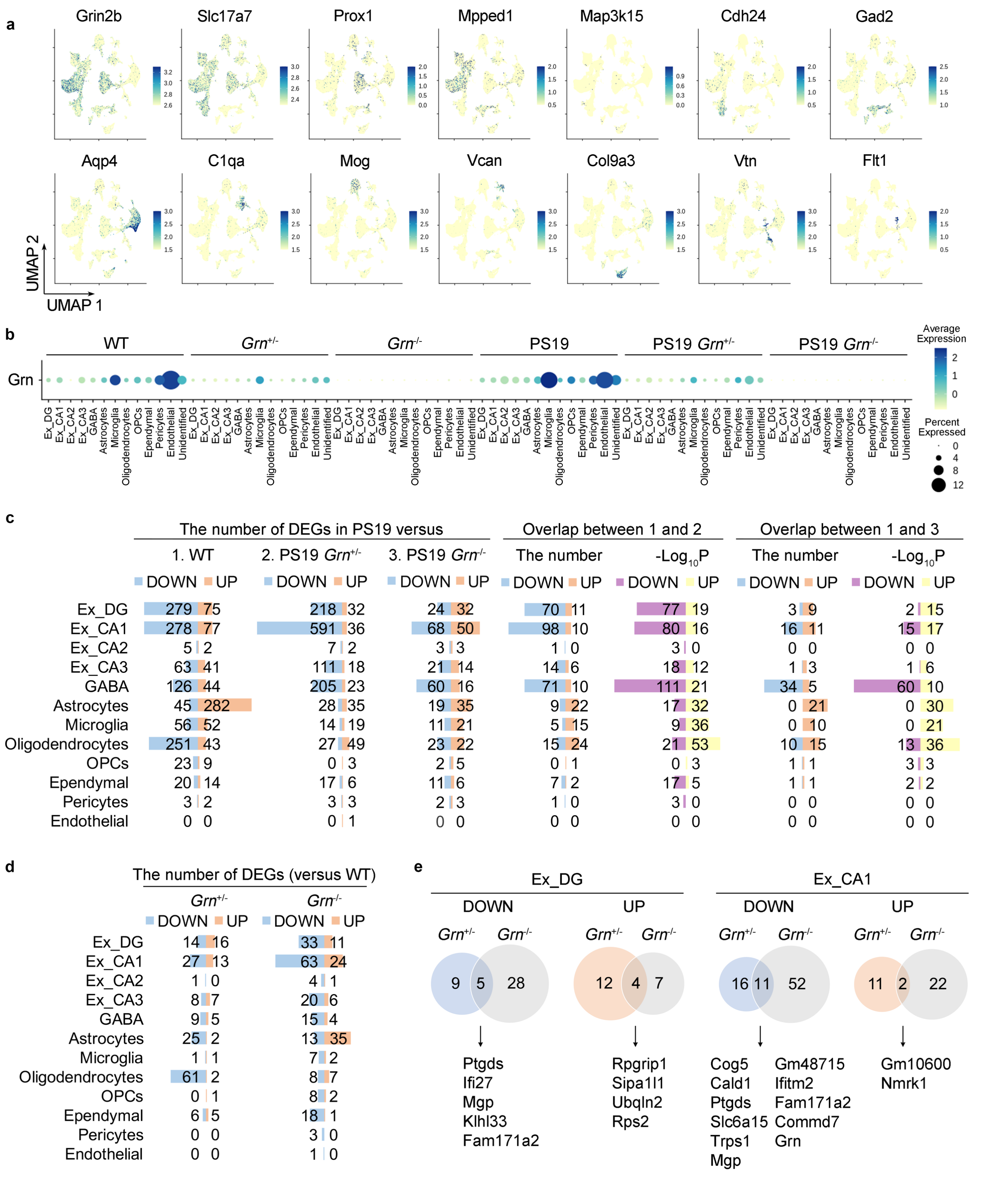

Supplementary Fig. 6: Marker gene expression and differential gene expression analyses in *Grn*$^{+/-}$ or *Grn*$^{-/-}$ vs. WT

a. UMAP plot colored by expression levels of marker genes: Grin2b (pan neurons), Slc17a7 (excitatory neurons), Prox1 (DG granule cells), Mpped1 (CA1 pyramidal cells), Map3k15 (CA2 pyramidal cells), Cdh24 (CA3 pyramidal cells), Gad2 (GABAergic neurons), Aqp4

(astrocytes), C1qa (microglia), Mog (oligodendrocytes), Vcan (oligodendrocytes precursor cells (OPCs)), Col9a3 (ependymal), Vtn (pericytes), Flt1 (endothelial).

b. Dot plot showing expression of Grn in each cell type of 6 genotypes.

c. Bar graphs showing the number of differentially-expressed genes (DEGs) (defined by Wilcoxon-rank-sum test, adjusted $p < 0.01$, $|\log(FC)| > 0.25$) in PS19 nuclei compared to WT, PS19 *Grn*$^{+/-}$, or PS19 *Grn*$^{-/-}$ nuclei, and the number of overlapping genes between DEGs from PS19 vs. WT and from PS19 vs. PS19 *Grn*$^{+/-}$ or from PS19 vs. PS19 *Grn*$^{-/-}$. Overlapping p-values ($-\log_{10}P$) by Fisher's exact test of each overlap are also bar-graphed.

d. Bar graphs showing the number of differentially-expressed genes (DEGs) in *Grn*$^{+/-}$ or *Grn*$^{-/-}$ nuclei compared to WT nuclei (Wilcoxon-rank-sum test, adjusted $p < 0.01$, $|\log(FC)| > 0.25$).

e. Overlaps of DEGs in Ex_DG and Ex_CA1 between *Grn*$^{+/-}$ and *Grn*$^{-/-}$ nuclei.

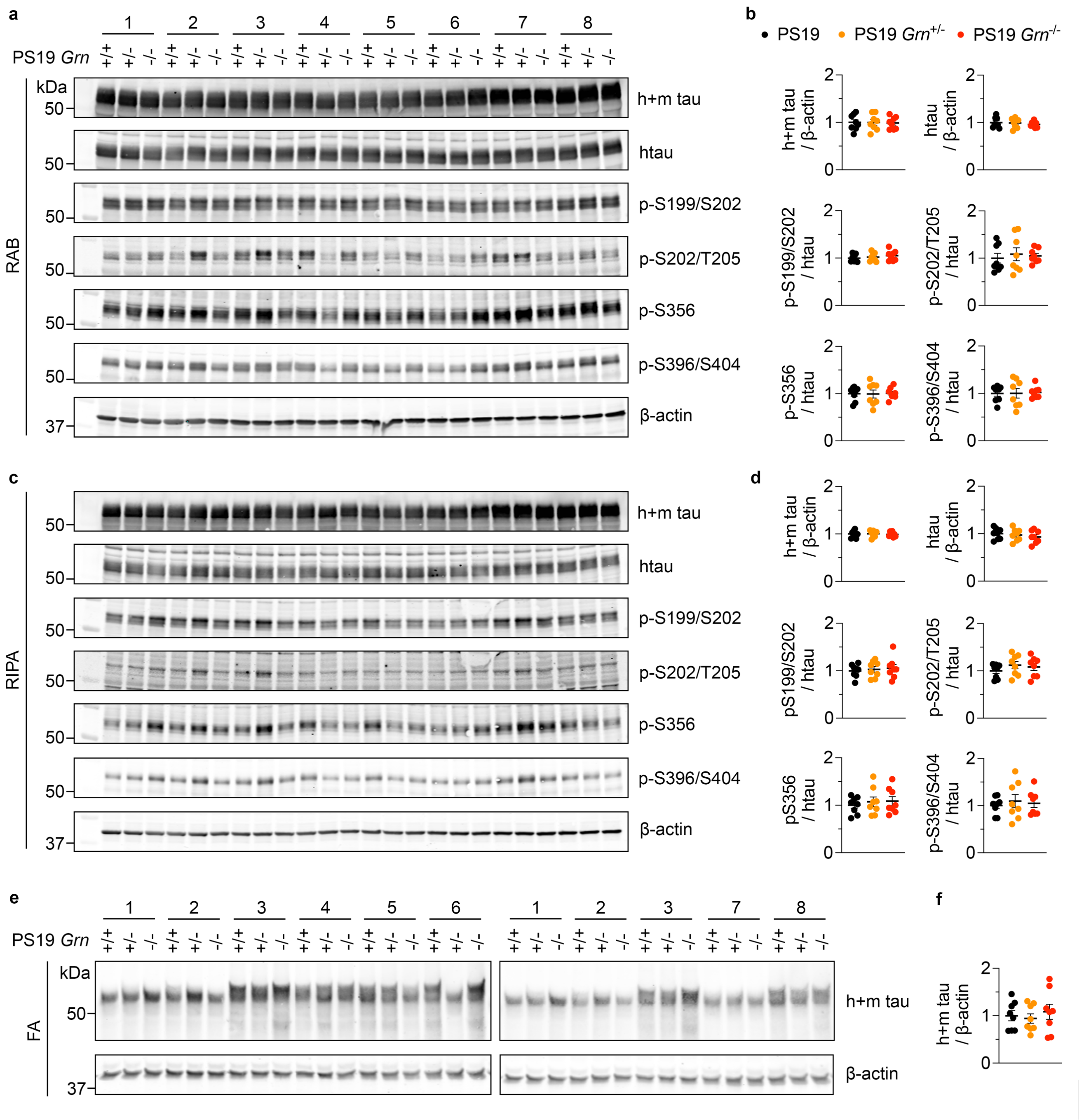

Supplementary Fig. 7: PGRN deficiency has no significant effects on tau phosphorylation in the RAB and RIPA-soluble fractions of PS19 mice

a. Immunoblot using anti-total tau, phospho-tau, and β-actin antibodies of the RAB-soluble fraction of 3 genotypes at 10 months of age.

b. Quantification of immunoblot in **a**. Mean ± SEM, n = 8 mice per genotype.

c. Immunoblot using anti-total tau, phospho-tau, and β-actin antibodies of the RIPA-soluble fraction of 3 genotypes at 10 months of age.

d. Quantification of immunoblot in **c**. Mean ± SEM, n = 8 mice per genotype.

e. Immunoblot using anti-total tau and β-actin antibodies of the FA-soluble fraction of 3 genotypes at 10 months of age.

f. Quantification of immunoblot in **e**. Mean ± SEM, n = 8 mice per genotype.

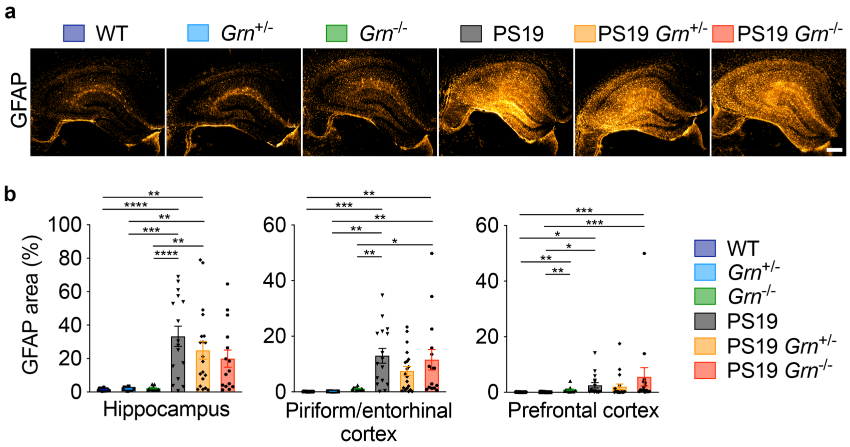

Supplementary Fig. 8: PGRN deficiency has no significant effects on astrogliosis

a. Representative images of GFAP staining of the hippocampus of 6 genotypes (WT, *Grn*$^{+/-}$, *Grn*$^{-/-}$, PS19, PS19 *Grn*$^{+/-}$, and PS19 *Grn*$^{-/-}$ mice) at 9-12 months of age. Bar, 300 μm.

b. Quantification of GFAP area (%) in 9-12-month-old 6 genotypes. Mean ± SEM, n = 14 mice (WT), n = 12 mice (*Grn*$^{+/-}$), n = 13 mice (*Grn*$^{-/-}$), n = 16 mice (PS19), n = 20 mice (PS19 *Grn*$^{+/-}$), and n = 15 mice (PS19 *Grn*$^{-/-}$), ****p = 0.00004 (hippocampus, WT vs. PS19), **p = 0.0025 (hippocampus, WT vs. PS19 *Grn*$^{+/-}$), ***p = 0.0001 (hippocampus, *Grn*$^{+/-}$ vs. PS19), **p = 0.0057 (hippocampus, *Grn*$^{+/-}$ vs. PS19 *Grn*$^{+/-}$), ****p = 0.00008 (hippocampus, *Grn*$^{-/-}$ vs. PS19), **p = 0.0050 (hippocampus, *Grn*$^{-/-}$ vs. PS19 *Grn*$^{+/-}$), ***p = 0.0006 (piriform/entorhinal cortex, WT vs. PS19), **p = 0.0037 (piriform/entorhinal cortex, WT vs. PS19 *Grn*$^{-/-}$), **p = 0.0012 (piriform/entorhinal cortex, *Grn*$^{+/-}$ vs. PS19), **p = 0.0065 (piriform/entorhinal cortex, *Grn*$^{+/-}$ vs. PS19 *Grn*$^{-/-}$), **p = 0.0025 (piriform/entorhinal cortex, *Grn*$^{-/-}$ vs. PS19), *p = 0.0133 (piriform/entorhinal cortex, *Grn*$^{-/-}$ vs. PS19 *Grn*$^{-/-}$), **p = 0.0093 (prefrontal cortex, WT vs. *Grn*$^{-/-}$), *p = 0.0361 (prefrontal cortex, WT vs. PS19), ***p = 0.0008 (prefrontal cortex, WT vs. PS19 *Grn*$^{-/-}$), **p = 0.0028 (prefrontal cortex, *Grn*$^{+/-}$ vs. *Grn*$^{-/-}$), *p = 0.0116 (prefrontal cortex, *Grn*$^{+/-}$ vs. PS19), ***p = 0.0002 (prefrontal cortex, *Grn*$^{+/-}$ vs. PS19 *Grn*$^{-/-}$), ****p < 0.0001; One-way ANOVA with Tukey's post hoc test or Kruskal-Wallis test with Dunn's post hoc test (for the prefrontal cortex).

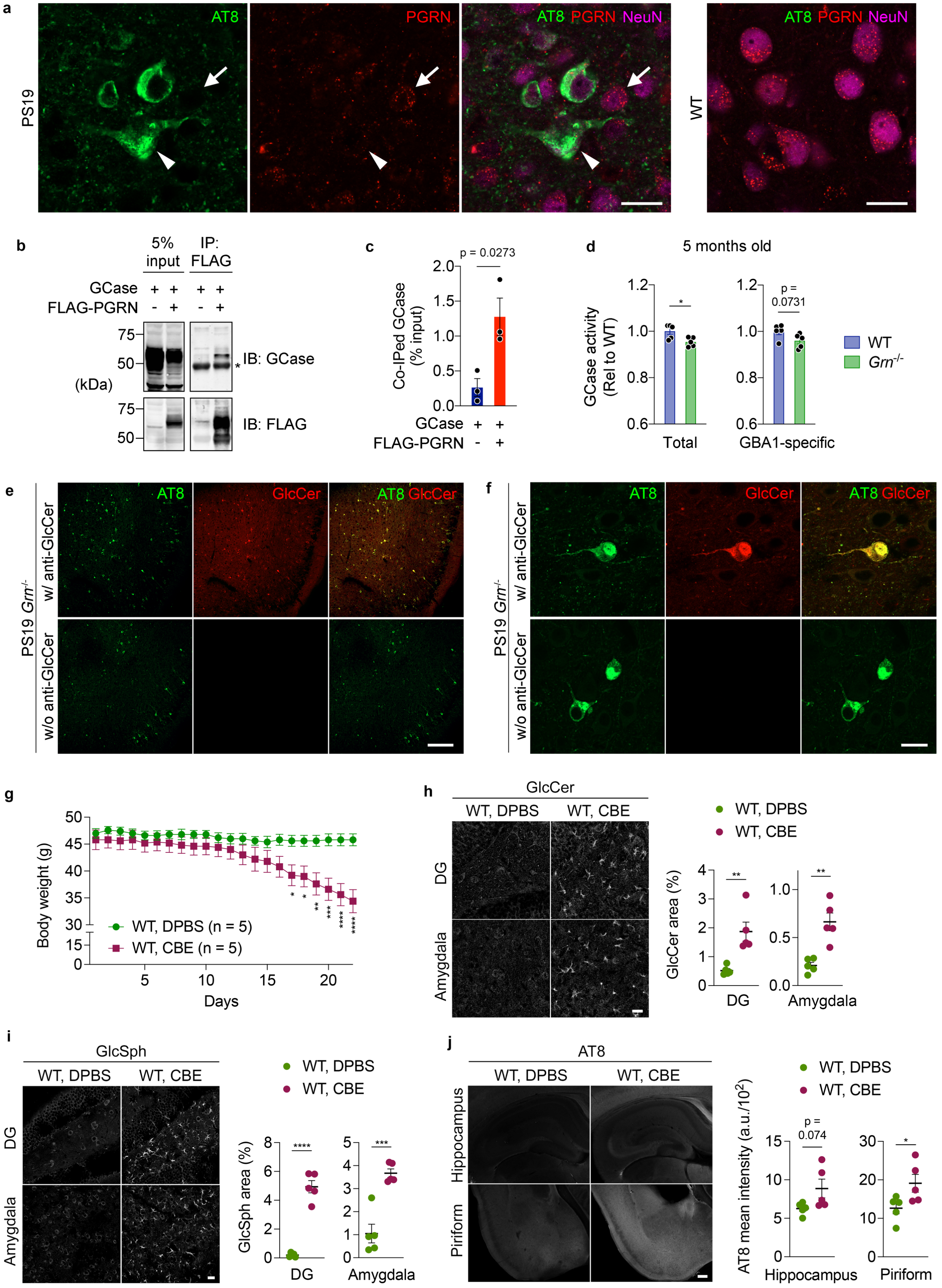
a
AT8
PGRN
AT8 PGRN NeuN
PS19
WT
b
5% input
IP: FLAG
GCase + + + +
FLAG-PGRN - + - +
75
50
(kDa)
IB: GCase
IB: FLAG
c
p = 0.0273
Co-IPed GCase (% input)
GCase + +
FLAG-PGRN - +
d
5 months old
GCase activity (Rel to WT)
p = 0.0731
Total
GBA1-specific
WT
Grn-/-
e
f
w/ anti-GlcCer
w/o anti-GlcCer
PS19 Grn-/-
GlcCer
AT8 GlcCer
g
Body weight (g)
Days
WT, DPBS (n = 5)
WT, CBE (n = 5)
h
GlcCer
WT, DPBS
WT, CBE
DG
Amygdala
GlcCer area (%)
i
GlcSph
GlcSph area (%)
j
AT8
Hippocampus
Piriform
AT8 mean intensity (a.u./10^2)
p = 0.074

Supplementary Fig. 9: Tau inclusions are positive for GlcCer in PS19 mice with PGRN reduction

a. Representative confocal images of AT8, PGRN, and NeuN co-staining in the amygdala region of PS19 and WT mice. Bar, 20 μm.

b. Co-IP assay using HEK293T cells co-expressing FLAG-PGRN and GCase suggesting interaction between PGRN and GCase.

c. Quantification of co-IP in **a**. Mean ± SEM, n = 3 experiments, p = 0.0273; Two-tailed unpaired t-test.

d. Total and GBA1-specific GCase activity of WT and *Grn*$^{-/-}$ cortices at 5 months of age. Mean ± SEM, n = 5 mice per genotype. *p = 0.0234; Two-tailed unpaired t-test.

e. Representative low-magnification confocal images of AT8 and GlcCer co-staining in the piriform/entorhinal cortex region of PS19 *Grn*$^{-/-}$ mice. In the bottom panels, the section was incubated only with AT8 antibody, followed by incubation with Alexa Fluor 488 and 568 secondary antibodies. All images were taken using the same setting. Bar, 200 μm.

f. Representative confocal images of GlcCer and AT8 co-staining in the amygdala region of PS19 *Grn*$^{-/-}$ mice. In the bottom panels, the section was incubated only with AT8 antibody, followed by incubation with Alexa Fluor 488 and 568 secondary antibodies. All images were taken using the same setting. Bar, 20 μm.

g. Body weight of 8-month-old WT mice injected with DPBS or 50 mg/kg CBE every day for 21 days. Mean ± SEM, n = 5 mice per genotype. *p = 0.0494 (Day 17), *p = 0.0363 (Day 18), **p = 0.0034 (Day 19), ***p = 0.0003, (Day 20), ****p = 0.00004 (Day 21), ****p = 0.000003 (Day 22); Two-way ANOVA with Sidak's post hoc test. There was interaction between two factors, time and treatment (p < 1e-15).

h. Representative images of GlcCer staining and quantification of GlcCer area (%) in the dentate gyrus (DG) and amygdala of 8-month-old WT treated with DPBS or CBE every day for 21 days. Mean ± SEM, n = 5 per groups, **p = 0.0039 (DG), **p = 0.0017 (amygdala); Two-tailed unpaired t-test.

i. Representative images of GlcSph staining and quantification of GlcSph area (%) in the dentate gyrus (DG) and amygdala of 8-month-old WT treated with DPBS or CBE every day for 21 days. Mean ± SEM, n = 5 per groups, ***p = 0.0004, ****p = 0.000004; Two-tailed unpaired t-test.

j. Representative images of AT8 staining and quantification of AT8 mean intensity in the hippocampus and piriform cortex of 8-month-old WT treated with DPBS or CBE every day for 21 days. Mean ± SEM, n = 5 per groups, *p = 0.0463; Two-tailed unpaired t-test.

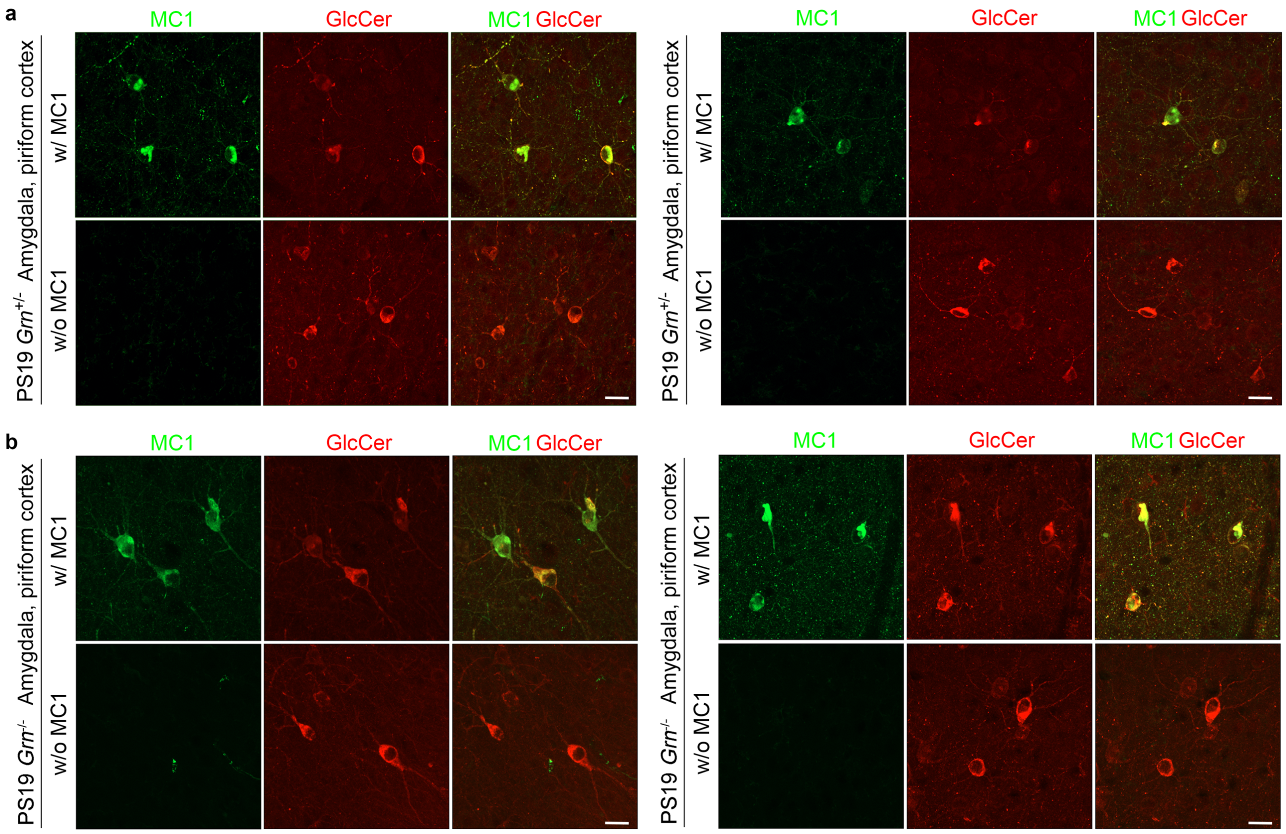

Supplementary Fig. 10: GlcCer-positive tau inclusions are labeled by MC1 antibody

a. Representative confocal images of MC1 and GlcCer co-staining in the amygdala and piriform cortex region of PS19 *Grn*$^{+/-}$ mice. In the bottom panels, the section was incubated only with anti-GlcCer antibody, followed by incubation with Alexa Fluor 488 and 568 secondary antibodies. Top and bottom images were taken using the same setting. Bar, 20 µm.

b. Representative confocal images of MC1 and GlcCer co-staining in the amygdala and piriform cortex region of PS19 *Grn*$^{-/-}$ mice. In the bottom panels, the section was incubated only with anti-GlcCer antibody, followed by incubation with Alexa Fluor 488 and 568 secondary antibodies. Top and bottom images were taken using the same setting. Bar, 20 µm.

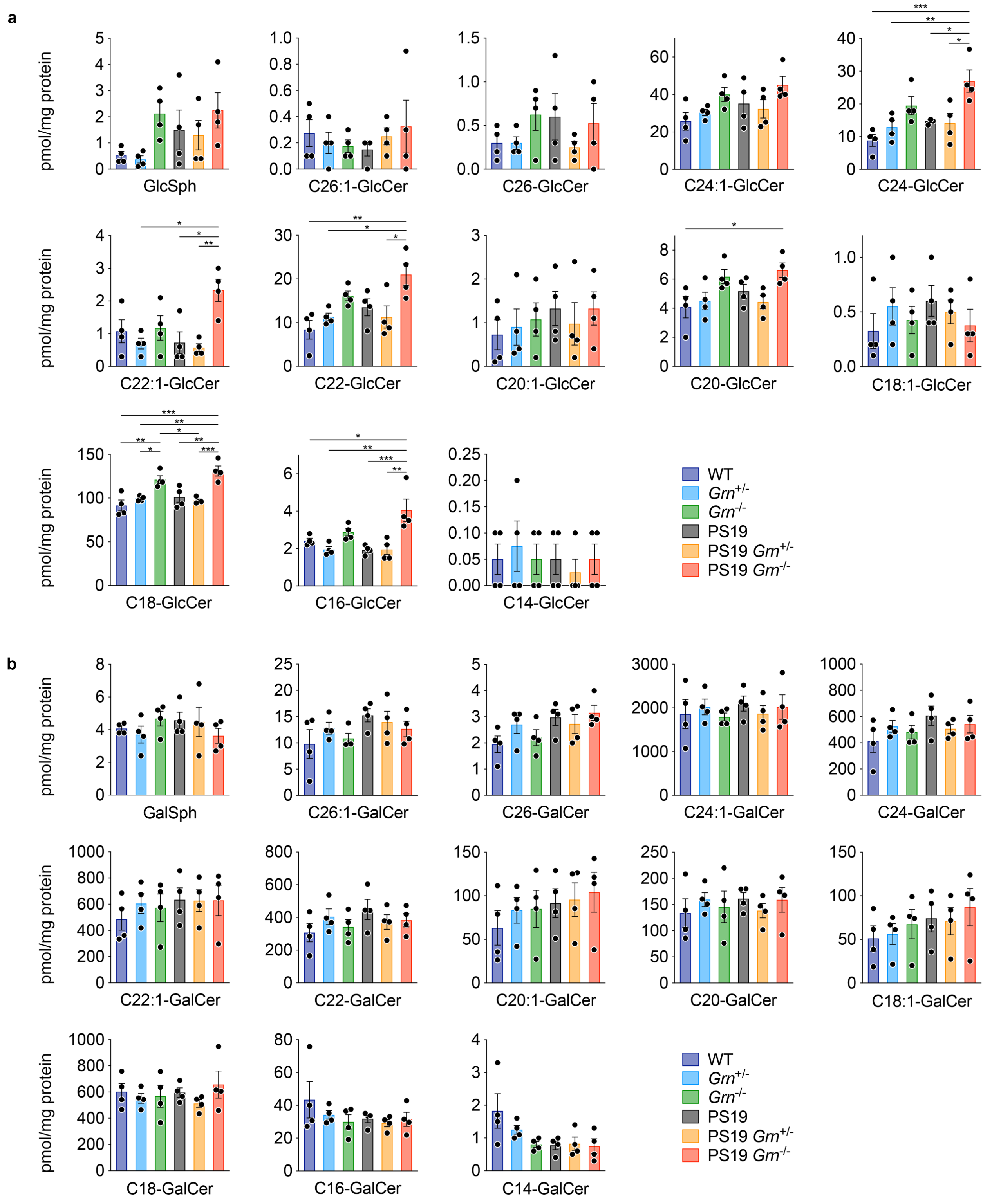

Supplementary Fig. 11: PGRN reduction increases GlcCer but not GalCer species

a. Levels of GlcCer species in the cortex of 6 genotypes at 10 months of age. Mean ± SEM, n = 4 mice per genotype. ***p = 0.0006 (C24-GlcCer, WT vs. PS19 *Grn*$^{-/-}$), **p = 0.0076 (C24-GlcCer, *Grn*$^{+/-}$ vs. PS19 *Grn*$^{-/-}$), *p = 0.0236 (C24-GlcCer, PS19 vs. PS19 *Grn*$^{-/-}$), *p = 0.0160 (C24-GlcCer, PS19 *Grn*$^{+/-}$ vs. PS19 *Grn*$^{-/-}$), *p = 0.0122 (C22:1-GlcCer, *Grn*$^{+/-}$

vs. PS19 *Grn*$^{-/-}$), *p = 0.0138 (C22:1-GlcCer, PS19 vs. PS19 *Grn*$^{-/-}$), **p = 0.0065 (C22:1-GlcCer, PS19 *Grn*$^{+/-}$ vs. PS19 *Grn*$^{-/-}$), **p = 0.0031 (C22-GlcCer, WT vs. PS19 *Grn*$^{-/-}$), *p = 0.0275 (C22-GlcCer, *Grn*$^{+/-}$ vs. PS19 *Grn*$^{-/-}$), *p = 0.0270 (C22-GlcCer, PS19 *Grn*$^{+/-}$ vs. PS19 *Grn*$^{-/-}$), *p = 0.0426 (C20-GlcCer, WT vs. PS19 *Grn*$^{-/-}$), **p = 0.0035 (C18-GlcCer, WT vs. *Grn*$^{-/-}$), ***p = 0.0002 (C18-GlcCer, WT vs. PS19 *Grn*$^{-/-}$), *p = 0.0416 (C18-GlcCer, *Grn*$^{+/-}$ vs. *Grn*$^{-/-}$), **p = 0.0018 (C18-GlcCer, *Grn*$^{+/-}$ vs. PS19 *Grn*$^{-/-}$), *p = 0.0205 (C18-GlcCer, *Grn*$^{-/-}$ vs. PS19 *Grn*$^{-/-}$), **p = 0.0032 (C18-GlcCer, PS19 vs. PS19 *Grn*$^{-/-}$), ***p = 0.0009 (C18-GlcCer, PS19 *Grn*$^{+/-}$ vs. PS19 *Grn*$^{-/-}$); *p = 0.0117 (C16-GlcCer, WT vs. PS19 *Grn*$^{-/-}$), **p = 0.0011 (C16-GlcCer, *Grn*$^{+/-}$ vs. PS19 *Grn*$^{-/-}$), ***p = 0.0009 (C16-GlcCer, PS19 vs. PS19 *Grn*$^{-/-}$), **p = 0.0011 (C16-GlcCer, PS19 *Grn*$^{+/-}$ vs. PS19 *Grn*$^{-/-}$); One-way ANOVA with Tukey's post hoc test.

b. Levels of GalCer species in the cortex of 6 genotypes at 10 months of age. Mean ± SEM, n = 4 mice per genotype. No significant difference was detected between 6 genotypes by one-way ANOVA.

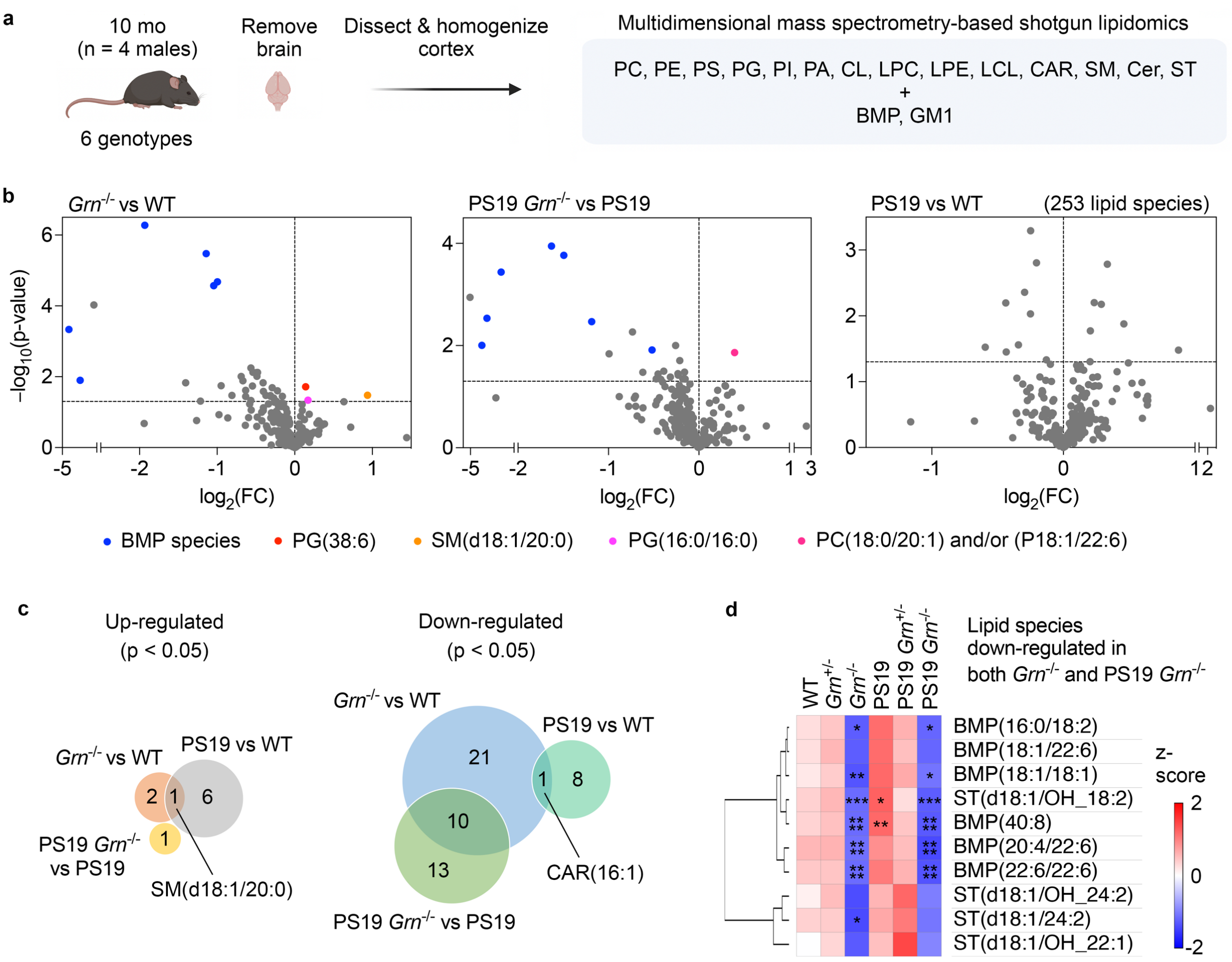

Supplementary Fig. 12: Lipidomic analysis of the cortex from 6 genotypes

a. Diagram showing the experimental procedures of lipidomic analysis using the cortex of 10-month-old 6 genotypes. n = 4 males per genotype. Multidimensional mass spectrometry-based shotgun lipidomics were performed to analyze 16 general lipid classes; phosphatidylcholine (PC), phosphatidylethanolamine (PE), phosphatidylserine (PS), phosphatidylglycerol (PG), phosphatidylinositol (PI), phosphatidic acid (PA), cardiolipin (CL), lyso-phosphatidylcholine (LPC), lyso-phosphatidylethanolamine (LPE), lyso-cardiolipin (LCL), carnitine (CAR), sphingomyelin (SM), ceramide (Cer), sulfatide (ST), bis(monoacylglycerol)phosphate (BMP), ganglioside GM1. The total number of lipid species analyzed in this study is 253. The diagram was created with BioRender.com.

b. Volcano plot showing all 253 molecular lipid species. BMP species ($p < 0.05$) are highlighted in blue. The horizontal line indicates p = 0.05. P values were calculated using two-tailed unpaired t-test.

c. Venn diagrams show the overlap between differentially regulated lipid species ($p < 0.05$) in *Grn*$^{-/-}$ (vs. WT), PS19 *Grn*$^{-/-}$ (vs. PS19), and PS19 (vs. WT) cortices.

d. Heatmap of all lipid species significantly down-regulated in both *Grn*$^{-/-}$ and PS19 *Grn*$^{-/-}$ cortices. n = 4 mice per genotype. *p = 0.0332 (BMP(16:0/18:2), *Grn*$^{-/-}$), *p = 0.0436 (BMP(16:0/18:2), PS19 *Grn*$^{-/-}$), **p = 0.0082 (BMP(18:1/18:1), *Grn*$^{-/-}$), *p = 0.0291 (BMP(18:1/18:1), PS19 *Grn*$^{-/-}$), ***p = 0.0004 (ST(d18:1/OH_18:2), *Grn*$^{-/-}$), *p = 0.0366

(ST(d18:1/OH_18:2), PS19), ***p = 0.0002 (ST(d18:1/OH_18:2), PS19 *Grn*$^{-/-}$), **p = 0.0055 (BMP(40:8), PS19), *p = 0.0162 (ST(d18:1/24:2), *Grn*$^{-/-}$), ****p < 0.0001; One-way ANOVA with Dunnett's post hoc test comparing to WT.

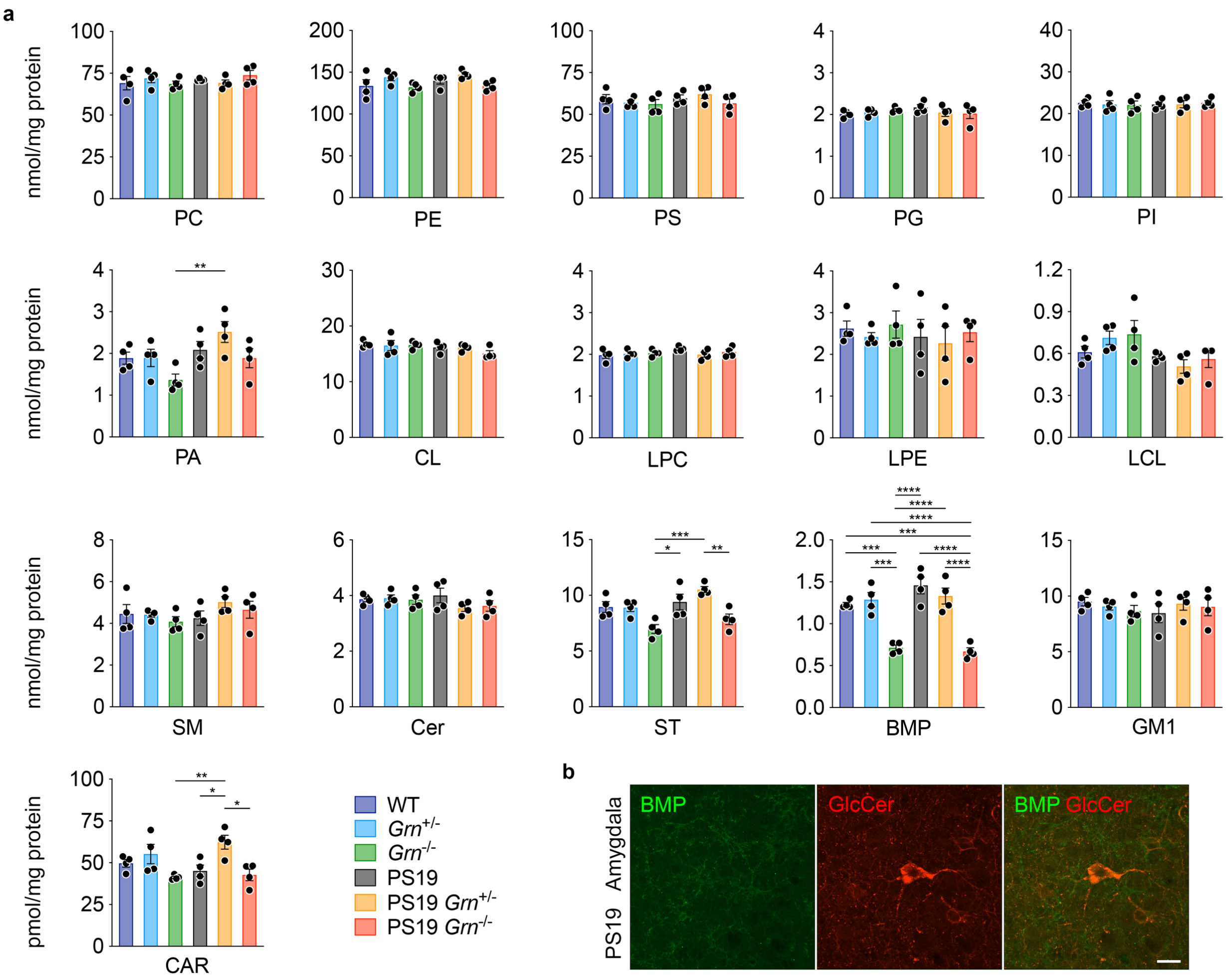

Supplementary Fig. 13: BMP levels are decreased in PGRN-deficient cortices

a. Total levels of 16 lipid classes (PC, PE, PS, PG, PI, PA, CL, LPC, LPE, LCL, SM, Cer, ST, BMP, GM1, CAR) in the cortex of 6 genotypes at 10 months of age. Mean ± SEM, n = 4 mice per genotype. *p = 0.0184 (ST, *Grn*$^{-/-}$ vs. PS19), ***p = 0.0005 (ST, *Grn*$^{-/-}$ vs. PS19 *Grn*$^{+/-}$), **p = 0.0084 (ST, PS19 *Grn*$^{+/-}$ vs. PS19 *Grn*$^{-/-}$), ***p = 0.0008 (BMP, WT vs. *Grn*$^{-/-}$), ***p = 0.0003 (BMP, WT vs. PS19 *Grn*$^{-/-}$), ***p = 0.0002 (BMP, *Grn*$^{+/-}$ vs. *Grn*$^{-/-}$), ****p = 0.0001 (BMP, *Grn*$^{+/-}$ vs. PS19 *Grn*$^{-/-}$), ****p = 0.00001 (BMP, *Grn*$^{-/-}$ vs. PS19), ****p = 0.0001 (BMP, *Grn*$^{-/-}$ vs. PS19 *Grn*$^{+/-}$), ****p = 0.000004 (BMP, PS19 vs. PS19 *Grn*$^{-/-}$), ****p = 0.00004 (BMP, PS19 *Grn*$^{+/-}$ vs. PS19 *Grn*$^{-/-}$), **p = 0.0081 (CAR, *Grn*$^{-/-}$ vs. PS19 *Grn*$^{+/-}$), *p = 0.0382 (CAR, PS19 vs. PS19 *Grn*$^{+/-}$), *p = 0.0149 (CAR, PS19 *Grn*$^{+/-}$ vs. PS19 *Grn*$^{-/-}$); One-way ANOVA with Tukey's post hoc test.

b. A representative confocal image of BMP and GlcCer co-staining in the amygdala of PS19 mice. Bar, 20 µm.

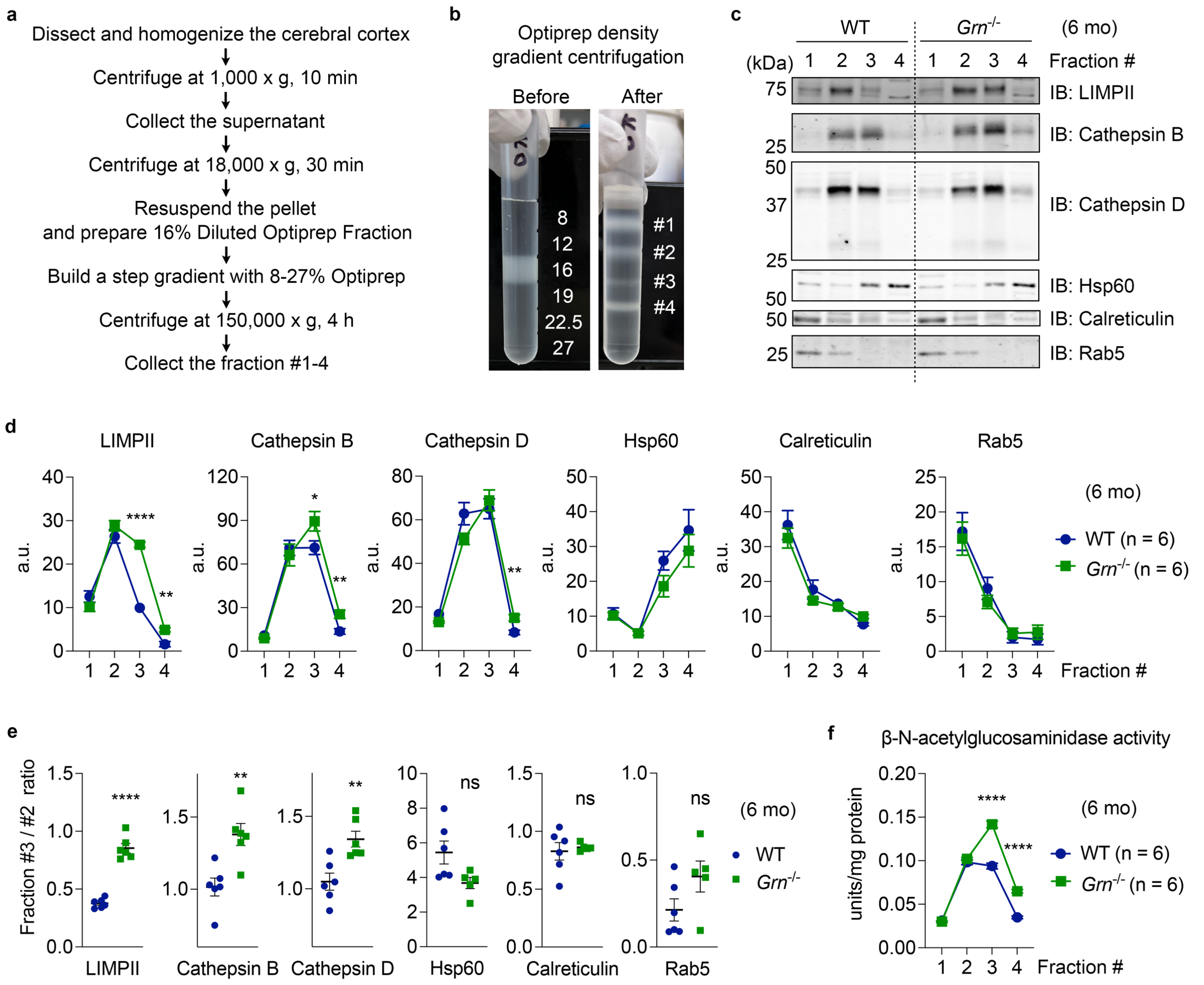

Supplementary Fig. 14: Isolation of lysosome-enriched fractions from 6-month-old WT and *Grn*$^{-/-}$ cortices

a. Summary of an experimental procedure of the Optiprep density gradient centrifugation for isolating lysosomes from mouse cortices. See Methods for more details.

b. Representative images of a sample tube before and after the Optiprep density gradient centrifugation.

c. Representative blots of the fraction #1-4 (1.5 μg protein) of 6-month-old WT and *Grn*$^{-/-}$ cortices using anti-LIMPII, cathepsin B, cathepsin D (lysosomal markers), Hsp60 (a mitochondria marker), calreticulin (an ER marker), and Rab5 (an early endosome marker).

d. Quantification of the blots in **c**. Mean ± SEM, n = 6 mice per genotype, ****p = 0.00000002 (LIMPII), **p = 0.0019 (LIMPII), *p = 0.0499 (cathepsin B), **p = 0.0056 (cathepsin B), **p = 0.0040 (cathepsin D); Two-tailed unpaired t-test.

e. Quantification of the fraction #3/#2 ratio of the blots in **c**. Mean ± SEM, n = 6 mice per genotype, ****p = 0.0000007 (LIMPII), **p = 0.0041 (cathepsin B), **p = 0.0045 (cathepsin D); Two-tailed unpaired t-test.

f. β-N-acetylglucosaminidase activity of the fractions #1-4 of 6-month-old WT and *Grn*$^{-/-}$ cortices. Mean ± SEM, n = 6 mice per genotype, ****p = 0.000003 (fraction #3), ****p = 0.0000004 (fraction #4); Two-tailed unpaired t-test.

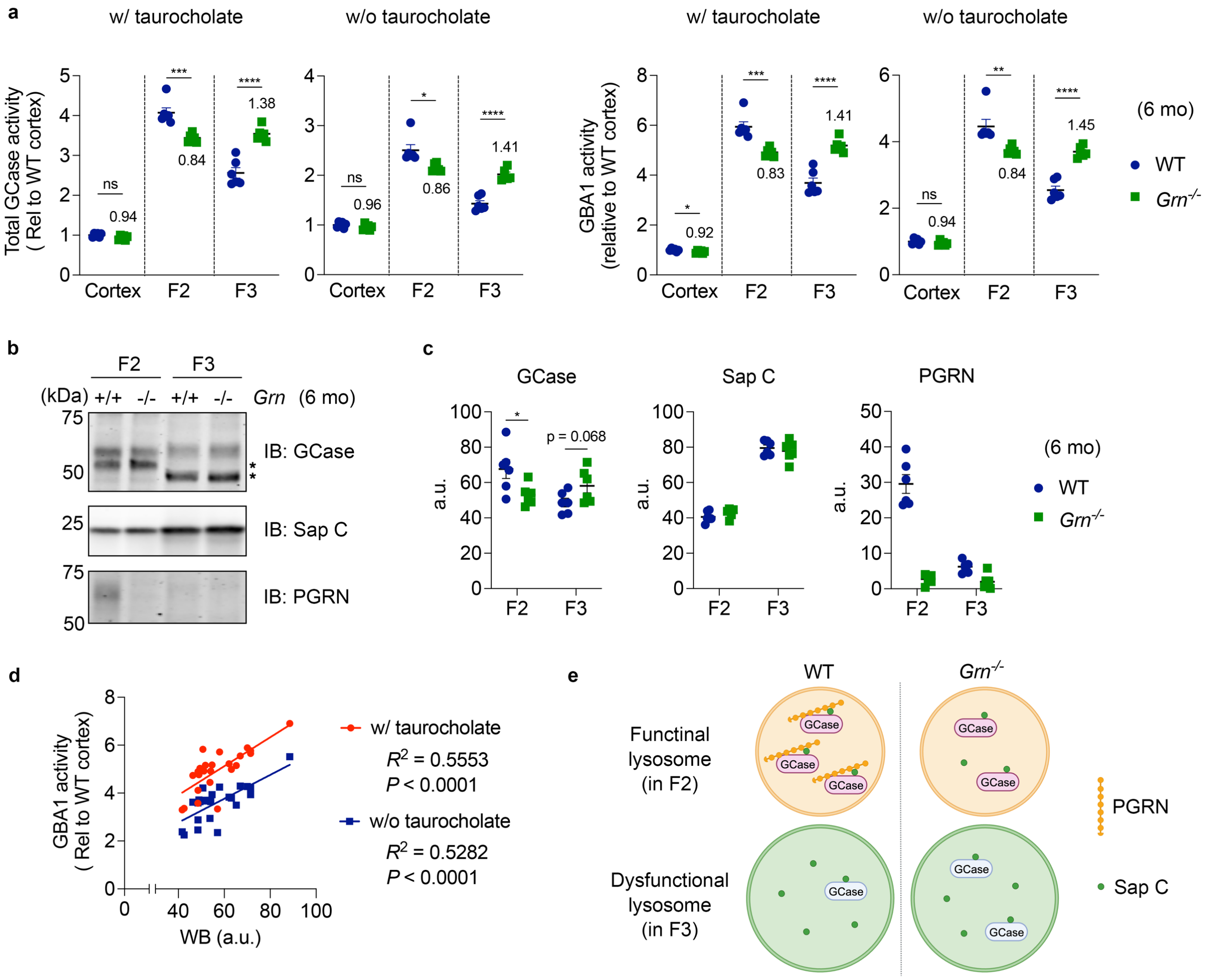

Supplementary Fig. 15: GCase activity of lysosome-enriched fractions from 6-month-old WT and *Grn*$^{-/-}$ cortices

a. Total and GBA1-specific GCase activity of the cortex and the lysosome-enriched fractions #3 and #4 of 6-month-old WT and *Grn*$^{-/-}$ mice. The GCase activity assay was performed with or without taurocholate as indicated. The fold change from WT for each sample was described in the graph. Mean ± SEM, n = 6 mice per genotype. ***p = 0.0006 (w/ taurocholate, total, F2), ****p = 0.00006 (w/ taurocholate, total, F3), *p = 0.0117 (w/o taurocholate, total, F2), ****p = 0.00001 (w/o taurocholate, total, F3), *p = 0.0117 (w/ taurocholate, GBA1-specific, cortex), ***p = 0.0006 (w/ taurocholate, GBA1-specific, F2), ****p = 0.00005 (w/ taurocholate, GBA1-specific, F3), **p = 0.0072 (w/o taurocholate, GBA1-specific, F2), ****p = 0.000007 (w/o taurocholate, GBA1-specific, F3); Two-tailed unpaired t-test.

b. Representative blots of the lysosome-enriched fractions #3 and #4 (7 μg protein) from 6-month-pld WT and *Grn*$^{-/-}$ cortices using anti-GCase, Saposin C (Sap C), and PGRN antibodies. The asterisks indicate non-specific bands that show up only when the membrane was overexposed with the primary antibody.

c. Quantification of the blots in **b**. Mean ± SEM, n = 6 mice per genotype, *p = 0.0309; Two-tailed unpaired t-test.

d. Correlation between the GCase-immunoreactive band intensity and GBA1-specific activity with or without taurocholate. n = 24 samples, p (two-tailed) = 0.00003 (w/ taurocholate), p (two-tailed) = 0.00006 (w/o taurocholate).

e. Hypothetical model of effects of PGRN deficiency on subcellular localization and activity of GCase. Two lysosomal proteins-enriched fractions #2 and #3 were found from the cortex of WT mice. In addition, PGRN deficiency caused an increase in accumulation of lysosomal markers in fraction #3. There was ~20% decrease in GCase activity in *Grn*$^{-/-}$ fraction #2, while PGRN deficiency increased GCase activity in fraction #3. Given the previous studies showing an increase in the size and number of lysosomes in *Grn*$^{-/-}$ brains and the increase in GlcCer levels in *Grn*$^{-/-}$ cortices (Fig. 5g and h), it is possible that enlarged dysfunctional lysosomes are accumulated in fraction #3 and that lysosomal enzymes in fraction #3 are not functional *in vivo*. PGRN deficiency had no significant effects on the lysosomal localization of Sap C, an activator of GCase. Taken together, PGRN deficiency may cause an increase in GlcCer levels not only by decreasing total amount of GCase in the cortex, but also by altering subcellular localization of GCase. The figure was created with BioRender.com.

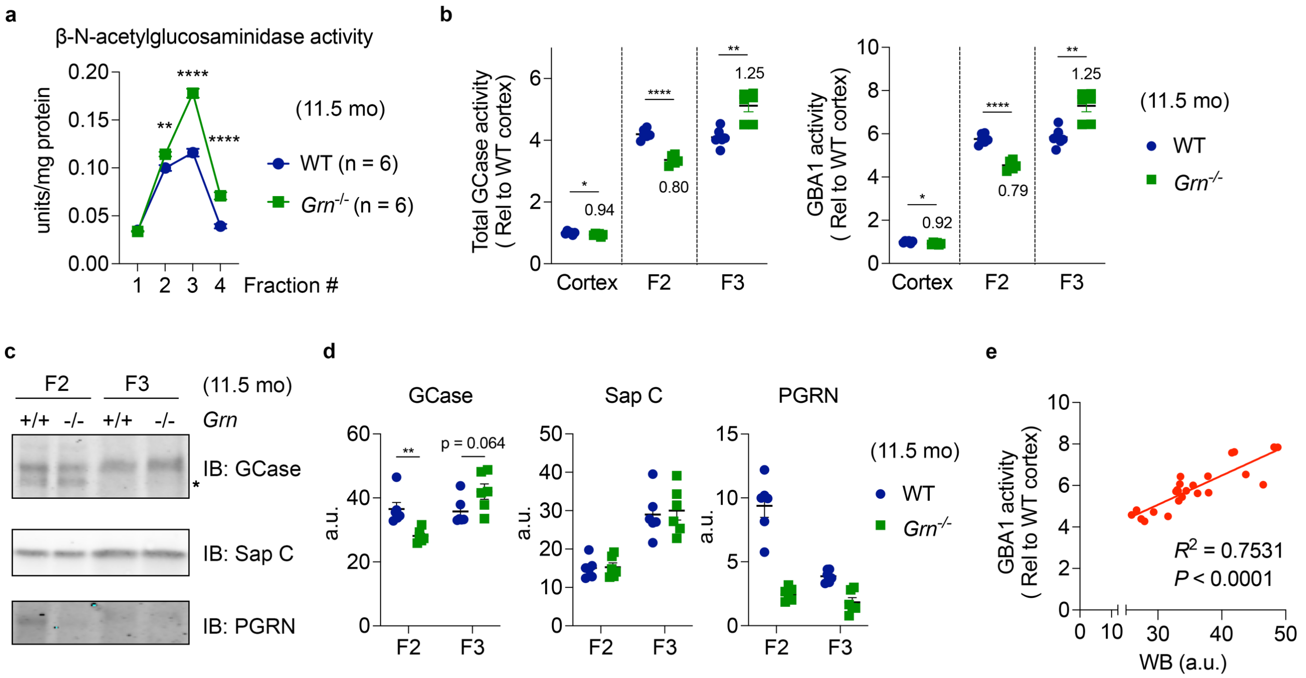

Supplementary Fig. 16: GCase activity of lysosome-enriched fractions from 11.5-month-old WT and *Grn*$^{-/-}$ cortices

a. β-N-acetylglucosaminidase activity of the fractions #1-4 of 11.5-month-old WT and *Grn*$^{-/-}$ cortices. Mean ± SEM, n = 6 mice per genotype, **p = 0.0040 (fraction #2), ****p = 0.000001 (fraction #3), ****p = 0.00003 (fraction #4); Two-tailed unpaired t-test.

b. Total and GBA1-specific GCase activity of the cortex and the lysosome-enriched fractions #3 and #4 of 11.5-month-old WT and *Grn*$^{-/-}$ mice. The GCase activity assay was performed with taurocholate. The fold change from WT for each sample was described in the graph. Mean ± SEM, n = 6 mice per genotype. *p = 0.0415 (total, cortex), ****p = 0.000003 (total, F2), **p = 0.0012 (total, F3), *p = 0.0156 (GBA1-specific, cortex), ****p = 0.000002 (GBA1-specific, F2), **p = 0.0012 (GBA1-specific, F3); Two-tailed unpaired t-test.

c. Representative blots of the lysosome-enriched fractions #3 and #4 (5.5 μg protein) from 11.5-month-pld WT and *Grn*$^{-/-}$ cortices using anti-GCase, Saposin C (Sap C), and PGRN antibodies. The asterisk indicates a non-specific band that shows up only when the membrane was overexposed with the primary antibody.

d. Quantification of the blots in **c**. Mean ± SEM, n = 6 mice per genotype, **p = 0.0035; Two-tailed unpaired t-test.

e. Correlation between the GCase-immunoreactive band intensity and GBA1-specific activity in 11.5-month-old samples. n = 24 samples, p (two-tailed) = 0.00000004.

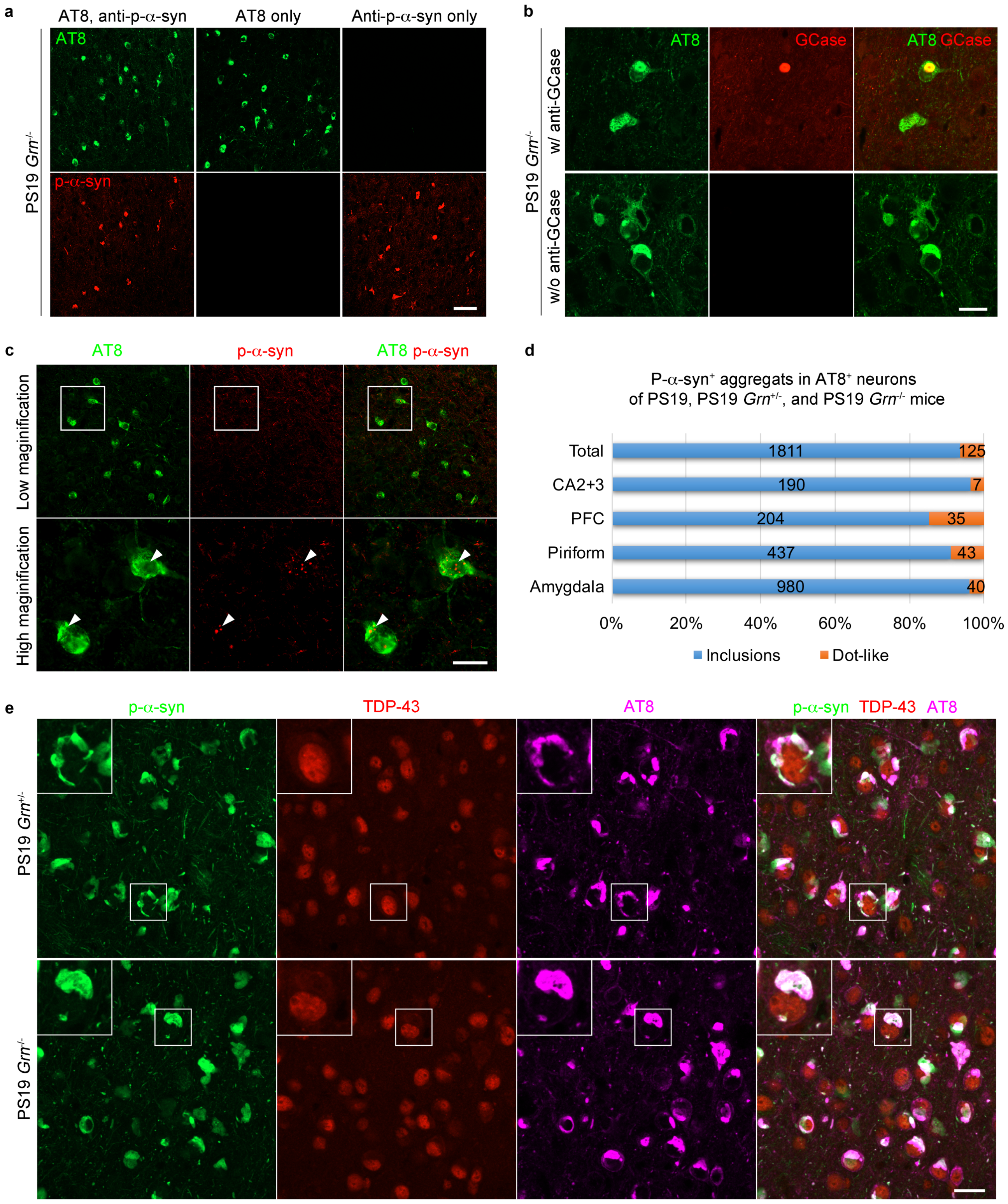

Supplementary Fig. 17: Co-localization of GlcCer and p-α-syn, but not TDP-43, is found in tau pathology of PS19 mice and human AD brains.

a. Representative confocal images of AT8 and p-α-syn co-staining in the amygdala region of PS19 *Grn*$^{-/-}$ mice. In the middle and right panels, the sections were incubated only with

AT8 or p-α-syn antibody, respectively, followed by incubation with Alexa Fluor 488 and 568 secondary antibodies. All images were taken using the same setting. Bar, 50 μm

b. Representative confocal images of AT8 and GCase co-staining of amygdala region of PS19 and PS19 *Grn*$^{-/-}$ mice. In the bottom panels, the section was incubated only with AT8 antibody, followed by incubation with Alexa Fluor 488 and 568 secondary antibodies. All images were taken using the same setting. Bar, 20 μm.

c. Representative confocal images of AT8 and p-α-syn co-staining in the amygdala of PS19 *Grn*$^{+/-}$ mice. The top panels were imaged using 20X lens, and the bottom ones were the white rectangular area imaged using 40X lens. Arrowheads indicate dot-like aggregates of p-α-syn in AT8-positive neurons. Bar, 20 μm.

d. The percentage of two types (inclusions and dot-like) of p-α-syn aggregates in AT8-positive neurons in each region of PS19 *Grn* cohorts. The total number of aggregates examined in each region was described in the bars.

e. Representative confocal images of p-α-syn, TDP-43, and AT8 triple staining in the amygdala of PS19 *Grn*$^{+/-}$ and PS19 *Grn*$^{-/-}$ mice. Bar, 20 μm.

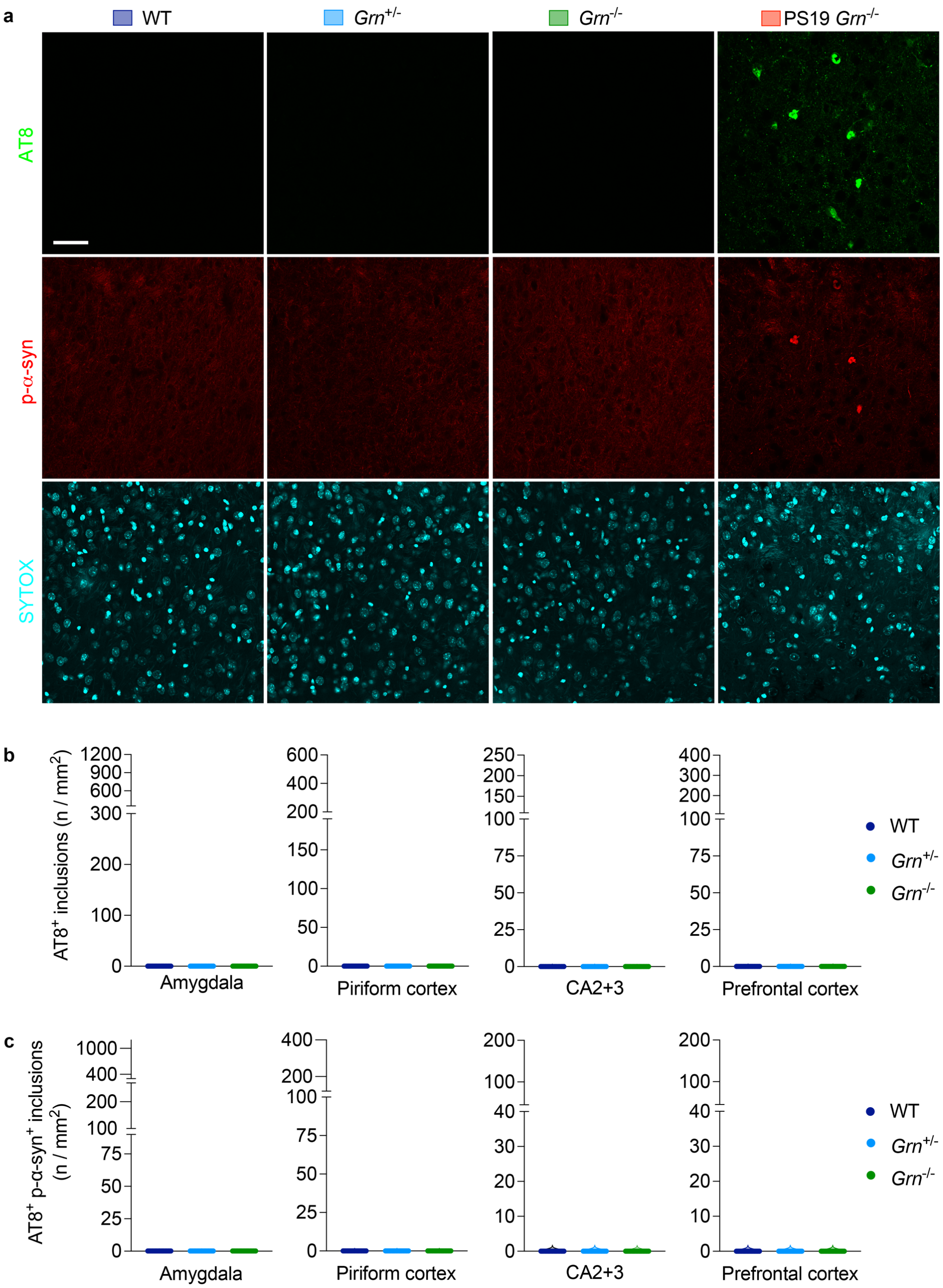

Supplementary Fig. 18: PGRN reduction does not cause tau and p-α-syn pathology in WT brains

a. Representative confocal images of AT8, p-α-syn, and SYTOX (nuclei) co-staining in the amygdala region of WT, *Grn*$^{+/-}$ and *Grn*$^{-/-}$ mice at 9-12 months of age. Brain sections from PS19 *Grn*$^{-/-}$ mice were used as a positive control. Bar, 50 μm.

b. Quantification of the number of AT8-positive tau inclusions of the amygdala, CA2 and CA3, piriform cortex, and prefrontal cortex of 3 genotypes (WT, *Grn*$^{+/-}$, and *Grn*$^{-/-}$ mice) at 10 months of age. Mean ± SEM, n = 6 mice per genotype.

c. Quantification of the number of AT8 and p-α-syn double positive inclusions of the amygdala, CA2 and CA3, piriform cortex, and prefrontal cortex of 3 genotypes (WT, *Grn*$^{+/-}$, and *Grn*$^{-/-}$ mice) at 10 months of age. Mean ± SEM, n = 6 mice per genotype.

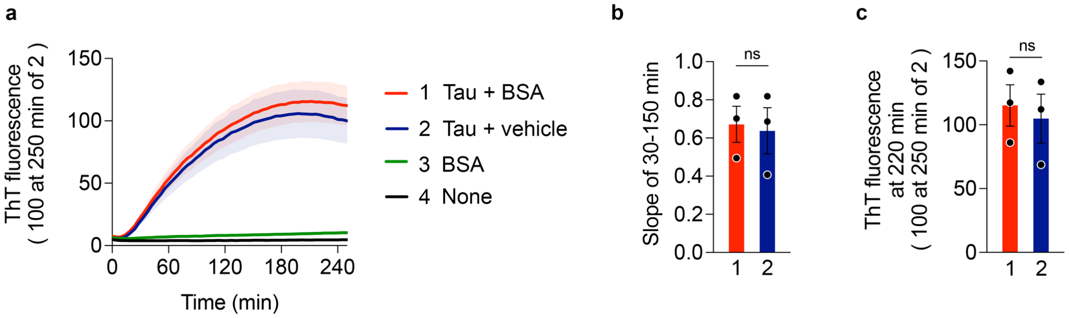

Supplementary Fig. 19: BSA has no significant effects on tau aggregation *in vitro*

a. ThT assay of heparin-induced aggregation of P301S tau in the presence or absence of BSA. Heparin was included in all samples except for the sample #4. Mean ± SEM, n = 3 experiments, each preformed in triplicate.

b. Slopes determined by linear regression from 30 to 150 min in **a**. Two-tailed paired t-test.

c. ThT fluorescence at 220 min in **a**. Two-tailed paired t-test.

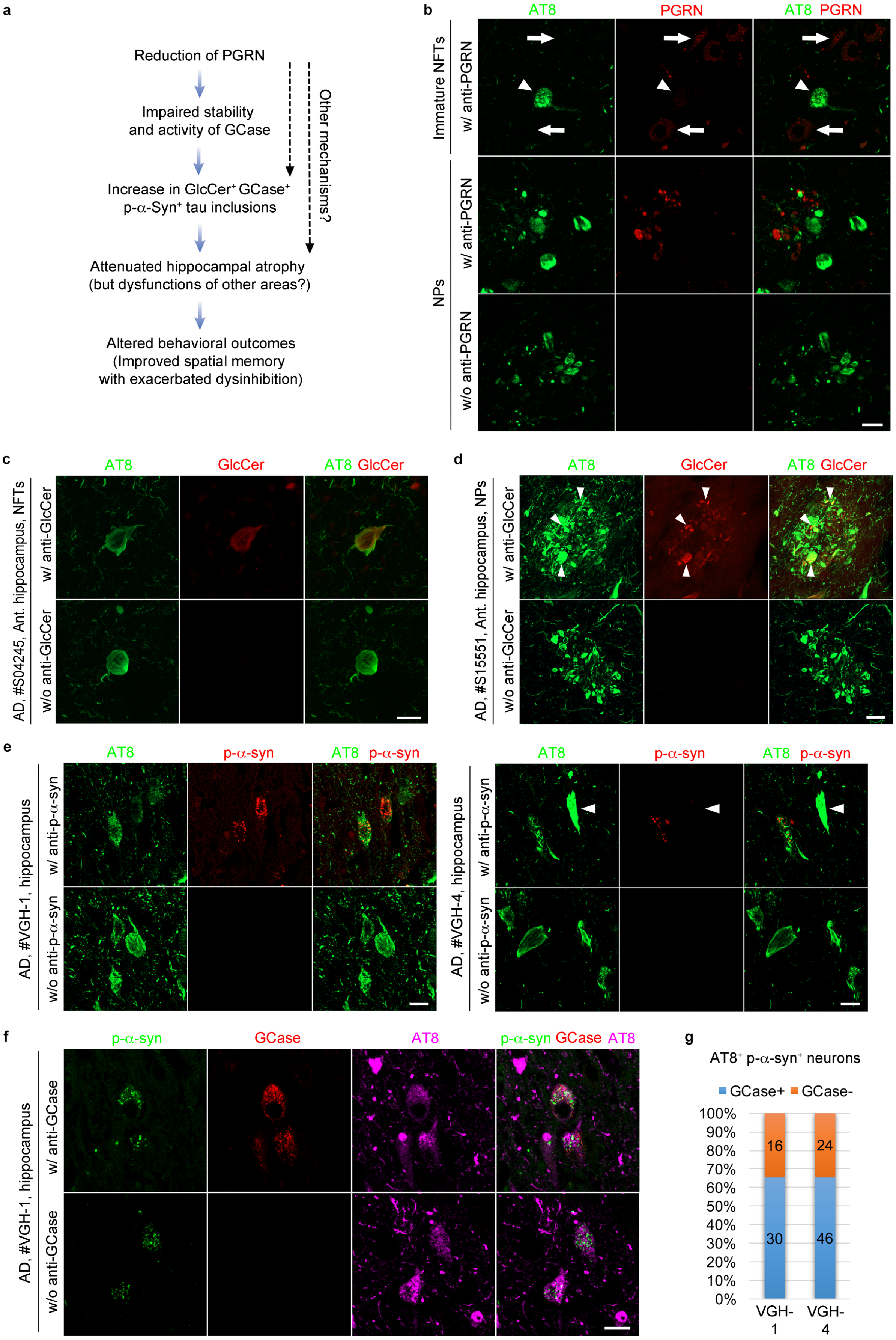
a
Reduction of PGRN
Impaired stability and activity of GCase
Increase in GlcCer+ GCase+ p-α-Syn+ tau inclusions
Attenuated hippocampal atrophy (but dysfunctions of other areas?)
Altered behavioral outcomes (Improved spatial memory with exacerbated dysinhibition)
Other mechanisms?
b
AT8
PGRN
AT8 PGRN
Immature NFTs
w/ anti-PGRN
NPs
w/ anti-PGRN
w/o anti-PGRN
c
AT8
GlcCer
AT8 GlcCer
AD, #S04245, Ant. hippocampus, NFTs
w/ anti-GlcCer
w/o anti-GlcCer
d
AT8
GlcCer
AT8 GlcCer
AD, #S15551, Ant. hippocampus, NPs
w/ anti-GlcCer
w/o anti-GlcCer
e
AT8
p-α-syn
AT8 p-α-syn
AD, #VGH-1, hippocampus
w/ anti-p-α-syn
w/o anti-p-α-syn
AD, #VGH-4, hippocampus
f
p-α-syn
GCase
AT8
p-α-syn GCase AT8
AD, #VGH-1, hippocampus
w/ anti-GCase
w/o anti-GCase
g
AT8+ p-α-syn+ neurons
GCase+
GCase-
100%
90%
80%
70%
60%
50%
40%
30%
20%
10%
0%
16
24
30
46
VGH-1
VGH-4

Supplementary Fig. 20: Co-localization of GCase and p-α-syn, but not PGRN, is found in tau pathology of human AD brains.

a. Hypothesis of effects of PGRN reduction in tauopathy. Reduction of PGRN causes impaired activity and stability of GCase, leading to an increase in $GlcCer^+$ p-α-syn^+ $GCase^+$ tau aggregates. Other mechanisms might be also involved in the increase of co-aggregation. The increase of co-aggregation attenuates hippocampal atrophy but might cause dysfunctions of other areas, leading to altered behavioral outcomes exhibiting exacerbated FTD-like disinhibition but improved AD-like spatial memory. It is possible that reduction of PGRN alters neurodegeneration and behaviors by other unknown mechanisms.

b. Representative confocal images of AT8 and PGRN co-staining of immature NFTs and NPs in the hippocampus of AD brains. Arrows indicate lysosomal PGRN in neurons. An arrowhead indicates an immature NFT with very low PGRN immunoreactivity. In the bottom panels, the section was incubated only with AT8 antibody, followed by incubation with Alexa Fluor 488 and 568 secondary antibodies. All images were taken using the same setting. Bar 20 μm.

c. Representative confocal images of AT8 and GlcCer co-staining of NFTs in the anterior hippocampus of AD patient ID#S04245. In the bottom panels, the section was incubated only with AT8 antibody, followed by incubation with Alexa Fluor 488 and 568 secondary antibodies. All images were taken using the same setting. Bar, 20 μm.

d. Representative confocal images of AT8 and GlcCer co-staining of NPs in the anterior hippocampus of AD patients ID#S15551. In the bottom panels, the section was incubated only with AT8 antibody, followed by incubation with Alexa Fluor 488 and 568 secondary antibodies. All images were taken using the same setting. Bar, 20 μm.

e. Representative confocal images of AT8 and p-α-syn co-staining of NFTs in the hippocampus of AD brain ID#VGH-1 and ID#VGH-4. An Arrowhead indicates a mature NFT with low p-α-syn immunoreactivity. In the bottom panels, the section was incubated only with AT8 antibody, followed by incubation with Alexa Fluor 488 and 568 secondary antibodies. All images were taken using the same setting. Bar, 20 μm.

f. Representative confocal images of AT8, GCase, and p-α-syn triple staining of NFTs in the hippocampus of AD brain ID#VGH-1. In the bottom panels, the section was incubated with AT8 and anti-p-α-syn antibodies but without anti-GCase antibody, followed by incubation with Alexa Fluor 488, 568, and 647 secondary antibodies. All images were taken using the same setting. Bar, 20 μm.

g. The percentage of GCase positivity in $AT8^+$ p-α-syn^+ neurons in AD brains #VGH-1 and #VGH-4. The number of aggregates examined is described in the bars.

Supplementary Fig. 21: Uncropped blots

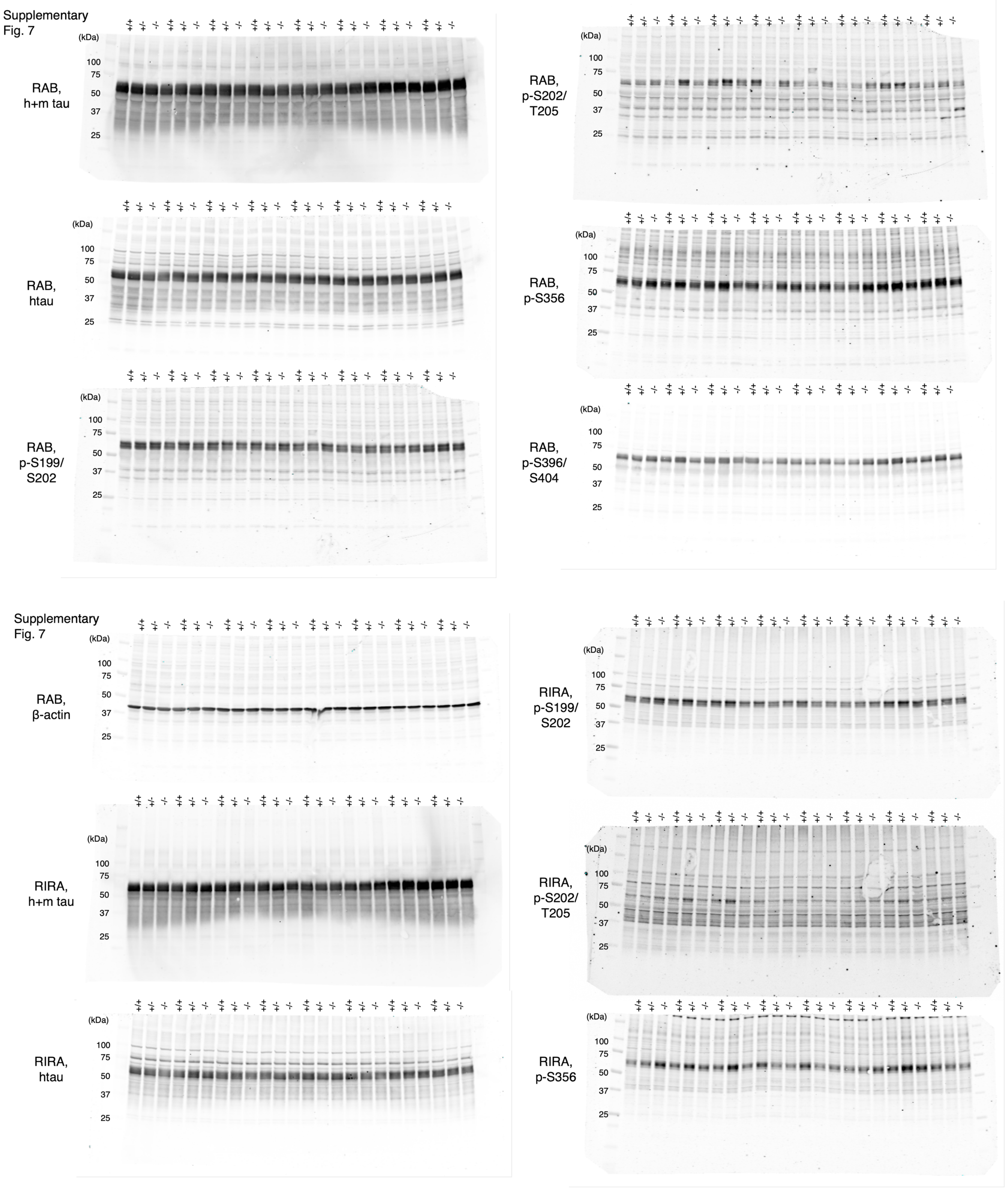

Supplementary Fig. 7

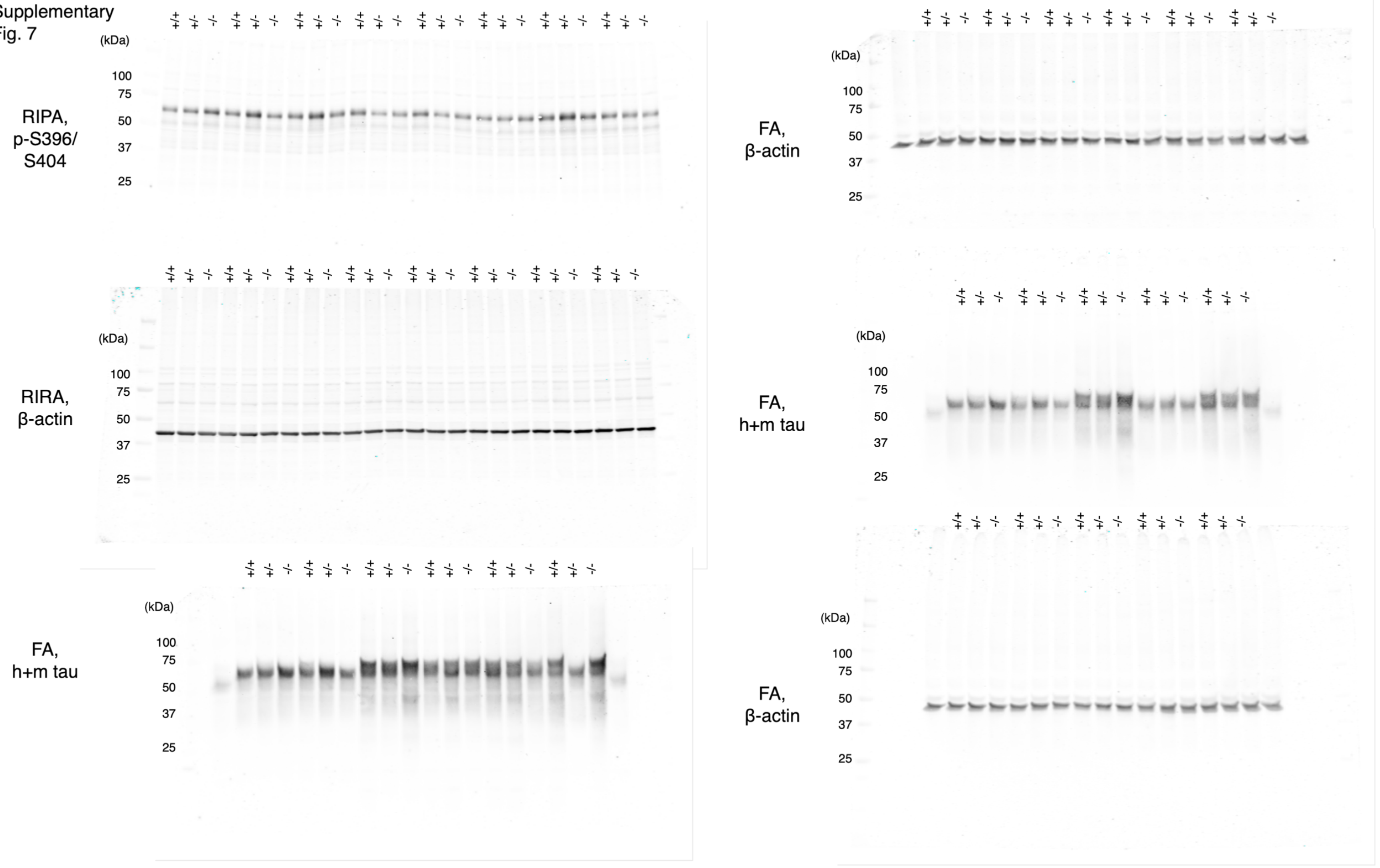

Supplementary Fig. 9b

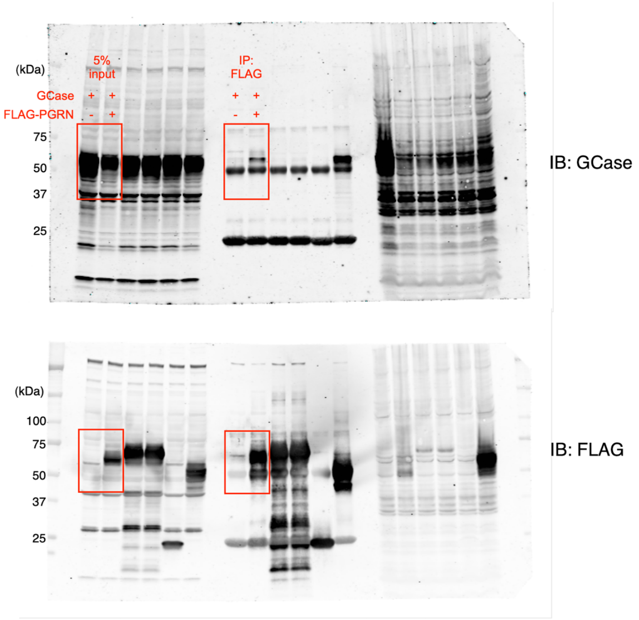

Supplementary Fig. 14c

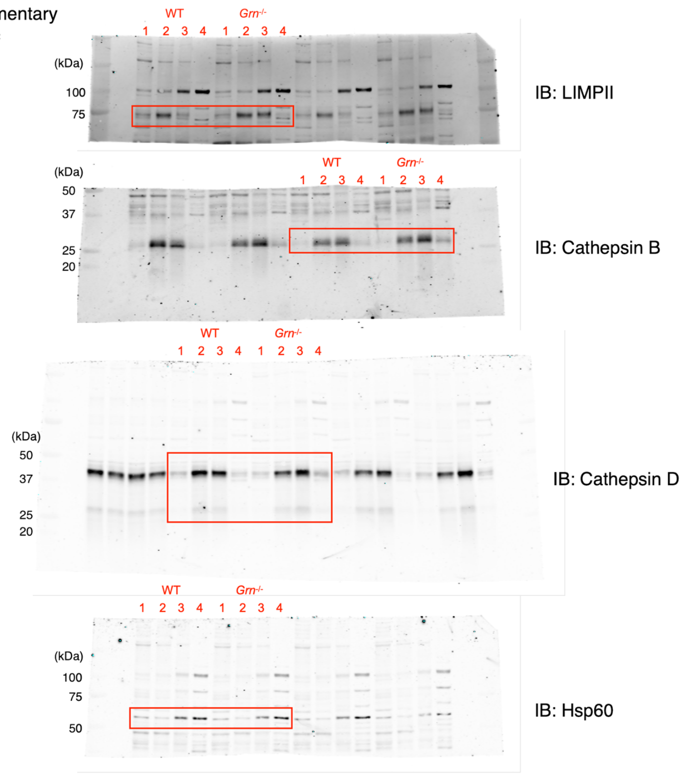

Supplementary Fig. 14c

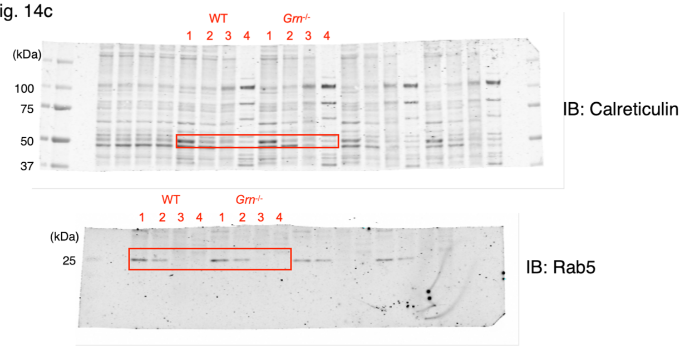

Supplementary Fig. 15b

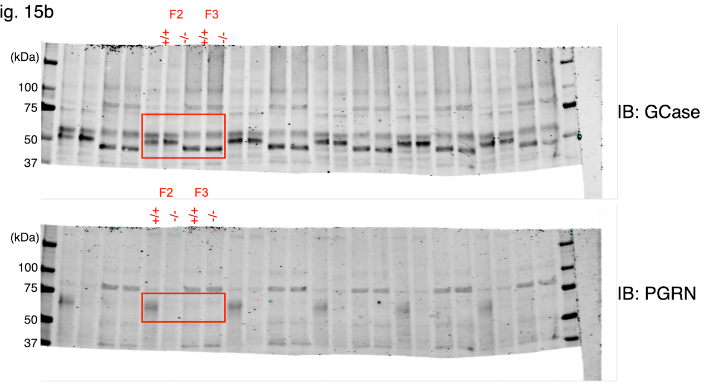

Supplementary Fig. 15b

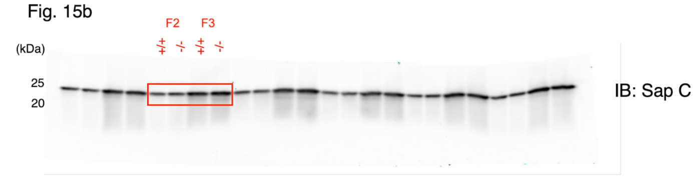

Supplementary Fig. 16c

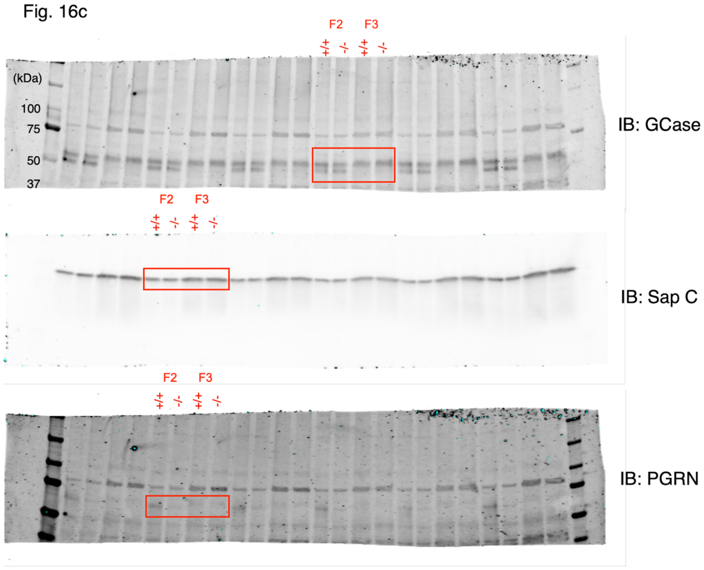

Supplementary Table 1. Demographic information for human samples used in this study

Source	Diagnosis	Sample (n)	Age (mean ± SD)	PMI (mean ± SD)	Braak
HBTRC	AD	5	78.40 ± 9.182	11.86 ± 8.917	VI or V
HBTRC	AH2	4	82.00 ± 19.20	14.37 ± 13.20	III or IV
HBTRC	DLB	4	73.75 ± 7.228	15.48 ± 8.086	0, I or II
VGH	AD	4	79.25 ± 8.884	42.00 ± 12.00	VI
VGH	FTD-GRN	2	81.00 ± 5.657	12.00 ± 0.000	II or III
Total samples used		20	78.30 ± 10.42	18.86 ± 14.92	

AH2; Alzheimer's changes moderate
HBTRC; Harvard brain tissue resource center
VGH; Vancouver General Hospital

Supplementary Table 2. Additional information for human samples used in this study

Brain ID	Diagnosis	Sex	Region examined	Section	Source
S05630	AD	F	Anterior hippocampus	Free-floating	HBTRC
S15551	AD	F	Anterior hippocampus	Free-floating	HBTRC
S15147	AD	F	Anterior hippocampus	Free-floating	HBTRC
S04245	AD	F	Anterior hippocampus	Free-floating	HBTRC
S19475	AD	M	Anterior hippocampus	Free-floating	HBTRC
S08380	AH2	M	Anterior hippocampus	Free-floating	HBTRC
S18596	AH2	F	Anterior hippocampus	Free-floating	HBTRC
S15386	AH2	M	Anterior hippocampus	Free-floating	HBTRC
S06423	AH2	M	Anterior hippocampus	Free-floating	HBTRC
S17240	DLB	M	Anterior hippocampus	Free-floating	HBTRC
S19496	DLB	M	Anterior hippocampus	Free-floating	HBTRC
S13303	DLB	M	Anterior hippocampus	Free-floating	HBTRC
S16963	DLB	M	Anterior hippocampus	Free-floating	HBTRC
13-78	FTD-GRN	F	Middle frontal gyrus	Free-floating	BSHRI
VGH-1	AD	F	Hippocampus	paraffine-embedded	VGH
VGH-2	AD	M	Hippocampus	paraffine-embedded	VGH
VGH-3	AD	F	Hippocampus	paraffine-embedded	VGH
VGH-4	AD	F	Hippocampus	paraffine-embedded	VGH
VGH-5	FTD-GRN	F	Hippocampus	paraffine-embedded	VGH
VGH-6	FTD-GRN	F	Hippocampus	paraffine-embedded	VGH

AH2; Alzheimer’s changes moderate
HBTRC; Harvard brain tissue resource center
BSHRI; Banner Sun Health Research Institute
VGH; Vancouver General Hospital
