# Supplementary material for: Reduced progranulin increases tau and α-synuclein inclusions and alters mouse tauopathy phenotypes via glucocerebrosidase
Source: Nat Commun. 2024 Feb 16;15:1434. doi: 10.1038/s41467-024-45692-3 (PMC10873339; doi:10.1038/s41467-024-45692-3)
Supplement: Supplementary file 3 — Description of Additional Supplementary Files [file 41467_2024_45692_MOESM3_ESM.pdf]

Description of Additional Supplementary Files

File Name: Supplementary Data 1
Description: Lipidomic analysis for GalCer and GluCer.

File Name: Supplementary Data 2
Description: Lipidomic analysis for general lipid panels, BMP, and GM1.
