# Supplementary material for: Reduced progranulin increases tau and α-synuclein inclusions and alters mouse tauopathy phenotypes via glucocerebrosidase
Source: Nat Commun. 2024 Feb 16;15:1434. doi: 10.1038/s41467-024-45692-3 (PMC10873339; doi:10.1038/s41467-024-45692-3)
Supplement: Supplementary file 6 — Reporting Summary [file 41467_2024_45692_MOESM6_ESM.pdf]

nature portfolio

Corresponding author(s): Stephen Strittmatter

Last updated by author(s): Feb 1, 2024

Reporting Summary

Nature Portfolio wishes to improve the reproducibility of the work that we publish. This form provides structure for consistency and transparency in reporting. For further information on Nature Portfolio policies, see our Editorial Policies and the Editorial Policy Checklist.

Statistics

For all statistical analyses, confirm that the following items are present in the figure legend, table legend, main text, or Methods section.

n/a	Confirmed	
[ ]	[x]	The exact sample size (*n*) for each experimental group/condition, given as a discrete number and unit of measurement
[ ]	[x]	A statement on whether measurements were taken from distinct samples or whether the same sample was measured repeatedly
[ ]	[x]	The statistical test(s) used AND whether they are one- or two-sided<br>*Only common tests should be described solely by name; describe more complex techniques in the Methods section.*
[x]	[ ]	A description of all covariates tested
[ ]	[x]	A description of any assumptions or corrections, such as tests of normality and adjustment for multiple comparisons
[ ]	[x]	A full description of the statistical parameters including central tendency (e.g. means) or other basic estimates (e.g. regression coefficient) AND variation (e.g. standard deviation) or associated estimates of uncertainty (e.g. confidence intervals)
[ ]	[x]	For null hypothesis testing, the test statistic (e.g. *F*, *t*, *r*) with confidence intervals, effect sizes, degrees of freedom and *P* value noted<br>*Give P values as exact values whenever suitable.*
[x]	[ ]	For Bayesian analysis, information on the choice of priors and Markov chain Monte Carlo settings
[x]	[ ]	For hierarchical and complex designs, identification of the appropriate level for tests and full reporting of outcomes
[ ]	[x]	Estimates of effect sizes (e.g. Cohen's *d*, Pearson's *r*), indicating how they were calculated

Our web collection on statistics for biologists contains articles on many of the points above.

Software and code

Policy information about availability of computer code

Data collection	Panlab SMART Video Tracking Software (Panlab, ver: 2.5.21),<br>ZEN 2.3 or 2.5 (ZEISS, blue edidtion),<br>MetaXpress (Molecular Device, ver 5.3.0.5)<br>PerkinElmer 2030 Manager (OerkinElmer, ver 4.0),<br>Odyssey Infrared Imaging system (LI-COR, ver 3.0.16),
Data analysis	Excel (Microsoft),<br>ImageJ(NIH),<br>Prism 9 (GraphPad),<br>Cell Ranger (10x Genomics, version: 3.0.2),<br>Seurat (version: 3.1.4), https://satijalab.org/seurat/,<br>ggplot2 (version: 3.3.0), https://ggplot2.tidyverse.org/,<br>DoubletFinder (version: 2.0.3), https://github.com/chris-mcginnis-ucsf/DoubletFinder,<br>Metascape, http://metascape.org/,<br>GeneOverlap, (version: 1.23.0) http://shenlab-sinai.github.io/shenlab-sinai/,<br>Morpheus, https://software.broadinstitute.org/morpheus,

For manuscripts utilizing custom algorithms or software that are central to the research but not yet described in published literature, software must be made available to editors and reviewers. We strongly encourage code deposition in a community repository (e.g. GitHub). See the Nature Portfolio guidelines for submitting code & software for further information.

Data

Policy information about availability of data

All manuscripts must include a data availability statement. This statement should provide the following information, where applicable:

- Accession codes, unique identifiers, or web links for publicly available datasets
- A description of any restrictions on data availability
- For clinical datasets or third party data, please ensure that the statement adheres to our policy

snRNA-seq raw data have been deposited at GEO (GSE180672). Mouse reference 3.0.0, mm10 was used (https://www.10xgenomics.com/support/software/cell-ranger/downloads/cr-ref-build-steps). Full results of lipidomic analysis are provided as Supplementary Data1 and 2. Source Data are provided with this paper.

Research involving human participants, their data, or biological material

Policy information about studies with human participants or human data. See also policy information about sex, gender (identity/presentation), and sexual orientation and race, ethnicity and racism.

Reporting on sex and gender	Sex of human brain samples is described in Supplementary Table 1.<br>Sex was not considered in the study design in Fig. 8 because only a small number of samples were available.
Reporting on race, ethnicity, or other socially relevant groupings	n/a
Population characteristics	Detailed characteristics of human brain tissues used in this study, including age, sex, diagnosis, and PMI, are described in Supplementary Table 1.
Recruitment	Samples of pre-existing human autopsy brain with no personal identifiers were obtained from Harvard Brain Tissue Resource Center, Banner Sun Health Research Institute, and Vancouver General Hospital.
Ethics oversight	There were no human subject participants. The human tissue was obtained from pre-existing autopsy tissue with no personal identifying information and deemed exempt from human subjects regulation by the Yale Institutional Review Board.

Note that full information on the approval of the study protocol must also be provided in the manuscript.

Field-specific reporting

Please select the one below that is the best fit for your research. If you are not sure, read the appropriate sections before making your selection.

☒ Life sciences ☐ Behavioural & social sciences ☐ Ecological, evolutionary & environmental sciences

For a reference copy of the document with all sections, see nature.com/documents/nr-reporting-summary-flat.pdf

Life sciences study design

All studies must disclose on these points even when the disclosure is negative.

Sample size	Sample size of experiments using mice was determined based on previous studies including ones using PS19 or Grn-/- mice (Spurrier et al., Sci Transl Med, 2022; Logan et al., Cell, 2021; Tang et al., Acta Neuropathol Commun, 2020; Zhou et al. PLoS One, 2019; Shi et al., JEM, 2019; Klein et al., Neuron, 2017; Shi et al., Nature, 2017; Takahashi et al., Acta Neuropathol, 2017; Leyns et al., PNAS, 2017). Sample size of AD-tau seeding assay was determined based on our previous studies (Tang et al., Acta Neuropathol Commun, 2020; Neis et al., JBC, 2021). Sample size of ThT assay was determined based on previous publications (Mazzulli et al., Cell, 2011; Briner et al., Cell Rep, 2020; Chakraborty et al., Nat Commun, 2021). Sample size of co-IP assay was determined based on our previous study (Klein et al. Neuron, 2017).
Data exclusions	In EPM test, outliers detected by ROUT (Q = 1%) were excluded because the animals were deemed to fail to be adapted to the test. In MWM, mice that took more than 30 s to reach the visible platform were excluded because they were deemed to have eye problems rather than memory problems or lack the motivation. The criteria were not pre-established, but similar criteria were used in our previous studies (Spurrier et al., Sci Transl Med, 2022; Tang et al., Acta Neuropathol Commun, 2020; Gunther et al., Cell Rep, 2019). No data were excluded in the other experiments.
Replication	For mouse experiments, all the biological replicates to support reproducibility are described in each figure legend. Mouse Morris water maze and elevated plus maze tests were performed with two independent cohorts and similar results were obtained. Immunohistochemical findings were verified by repeating the staining at least once with different antigen combinations in different experiments. Similar results were obtained in the replication staining experiments. Co-IP and GCase activity assays were performed to replicate previous publications as described in the manuscript. An increase in GlcCer levels in Grn-/- mice was confirmed by both lipidomics and immunohistochemistry. In addition, our results of lipidomic analysis are consistent with a previous publication (Logan et al., Cell, 2021). At least three independent experiments were performed in co-IP, AD-tau seeding, and Thioflavin T assays. At least two independent EM image sessions were conducted using independently prepared samples to confirm reproducibility. All attempts at replication were successful.
Randomization	Animals were grouped based on their genotype. Primary cultured neurons for AD-tau seeding assay and tau protein for ThT assay were randomly allocated into experimental groups.

Blinding

All behavioral tests, immunohistochemical, lipidomic, and electron microscopic data collection and analyses were performed by investigators who were blinded to the genotypes. Investigators were also blinded to the genotypes during data collection for single-nucleus RNA-sequencing experiments. Images of AD-tau seeding assay were automatically and unbiasedly collected using ImageXpress and the analysis was performed by an investigator blinded to the samples. Investigators were not blinded during nuclei isolation and lysosomal fractionation to minimize batch effects because the nuclei or lysosome preparation was repeated several days to complete all samples. Investigators were not blinded during the single-nucleus RNA-sequencing analysis using the Seurat package, because the data were analyzed based on the genotypes. In immunoblot and co-IP assay, investigators were unblinded to determine the loading order of the samples. Investigators were not blinded to the sample groups in GCase activity assay and Thioflavin T assay because the data were automatically collected with Victor 3 or VICOTR Nivo plate reader.

Reporting for specific materials, systems and methods

We require information from authors about some types of materials, experimental systems and methods used in many studies. Here, indicate whether each material, system or method listed is relevant to your study. If you are not sure if a list item applies to your research, read the appropriate section before selecting a response.

Materials & experimental systems

n/a	Involved in the study	
☐	☒	Antibodies
☐	☒	Eukaryotic cell lines
☒	☐	Palaeontology and archaeology
☐	☒	Animals and other organisms
☒	☐	Clinical data
☒	☐	Dual use research of concern
☒	☐	Plants

Methods

n/a	Involved in the study	
☒	☐	ChIP-seq
☒	☐	Flow cytometry
☒	☐	MRI-based neuroimaging

Antibodies

Antibodies used

Sheep anti-mouse PGRN (R&D systems, AF2557),
Goat anti-human PGRN (R&D systems, AF2420),
Mouse anti-phospho-tau 202/205 (AT8) (Invitrogen, MN1020),
Rabbit anti-tau (DAKO, A0024),
Mouse anti-tau (HT7) (Invitrogen, MN1000),
Mouse anti-phospho-tau 396/404 (PHF1) (gift from Dr. Peter Davies),
Mouse MC1 (gift from Dr. Peter Davies),
Rabbit anti-phospho-tau 199/202 (Invitrogen, 44-768G),
Rabbit anti-phospho-tau 356 (Invitrogen, 44-51G),
Mouse anti-beta-actin (Cell Signaling Technology, 3700),
Rabbit anti-Iba1 (FUJIFILM Wako, 0190-19741),
Rat anti-CD68 (Bio-rad, MCA1957),
Rabbit anti-GFAP (Abcam, Ab7260),
Rabbit anti-glucosylceramide (Glycobiotech, RAS_0011),
Rabbit anti-glucosylsphingosine (Antibody Research Corporation, 111584),
Rabbit anti-GCase (SIGMA, G4171),
Mouse anti-phospho-alpha-synuclein (81A) (BioLegend, MMS-5091),
Rabbit anti-TDP-43 (proteintech, 10782-2-AP),
Rabbit anti-NeuN (abcam, ab177487),
Rabbit anti-FLAG (SIGMA, F7425),
Mouse anti-mouse tau (T49) (SIGMA, MABN827),
Rabbit anti-MAP2 (Cell Signalling, 4542),
Mouse anti-BMP (Echelon #Z-PLBPA),
Rabbit anti-LIMPII (Novus Biologicals #NB400-129),
Goat anti-mouse cathepsin B (R&D #AF965),
Goat anti-mouse cathepsin D (R&D #AF1029),
Rabbit anti-Hsp60 (Cell signaling #4870),
Mouse anti-calreticulin (Novus Biologicals #681233),
Rabbit anti-Rab5 (Cell signaling #3547),
Mouse anti-Sap C (Santa Cruz #sc-347119),
Donkey anti-Mouse IgG (H+L), Alexa Fluor 488 (Invitrogen, A21202),
Donkey anti-Rabbit IgG (H+L), Alexa Fluor 568 (Invitrogen, A10042),
Donkey anti-Rabbit IgG (H+L), Alexa Fluor 647 (Invitrogen, A31573),
Donkey anti-Sheep IgG (H+L), Alexa Fluor 568 (Invitrogen, A21099),
Donkey anti-Sheep IgG (H+L), Alexa Fluor 488 (Invitrogen, A11015),
Donkey anti-Rat IgG (H+L), Alexa Fluor 488 (Invitrogen, A21208),

Donkey anti-Goat IgG (H+L), Alexa Fluor 568 (Invitrogen, A11057),
Goat anti-Mouse IgG1, Alexa Fluor 488 (Invitrogen, A21121),
Goat anti-Mouse IgG2a, Alexa Fluor 568 (Invitrogen, A21134),
Goat anti-Mouse IgG2a, Alexa Fluor 488 (Invitrogen, A21131),
Goat anti-Rabbit IgG (H+L), Alexa Fluor 568 (Invitrogen, A11036),
Goat anti-Mouse IgG1, Alexa Fluor 647 (Invitrogen, A21240),
Donkey IRDye 680LT anti-Mouse (LI-COR 926-68022),
Donkey IRDye 680LT anti-Rabbit (LI-COR 926-68023),
Donkey IRDye 800CW anti-Mouse (LI-COR 926-32212),
Donkey IRDye 800CW anti-Rabbit (LI-COR 926-32213),
Donkey IRDye 800CW anti-Goat (LI-COR 926-32214),
Donkey anti-Sheep IgG (H+L), Alexa Fluor 680 (Invitrogen, A21102),

Validation

The sheep anti-mouse PGRN antibody (R&D, #AF2557) has been cited at least 30 times (https://www.rndsystems.com/products/mouse-progranulin-pgrn-antibody_af2557). We also confirmed the specificity using Grn-/- mice in immunohistochemistry (Supplementary Fig.2 and western blotting (Supplementary Fig. 15 and 16) in the present study.

The goat anti-human PGRN antibody (R&D, #AF2420) has been cited at least 18 times and validated for use in both western blotting and immunohistochemisitry (https://www.rndsystems.com/products/human-progranulin-pgrn-antibody_af2420).

The mouse anti-phospho-tau 202/205 (AT8) antibody (Invitrogen, #MN1020) has long been commonly used in the field, cited at least 817 times, and validated for use in both western blotting and immunohistochemistry (https://www.thermofisher.com/antibody/product/Phospho-Tau-Ser202-Thr205-Antibody-clone-AT8-Monoclonal/MN1020).

The rabbit anti-tau antibody (DAKO, #A0024) has been cited at least 362 times and validated for use in western blotting (https://www.citeab.com/antibodies/3382933-a0024-tau).

The mouse anti-tau (HT7) antibody (Invitrogen, #MN1000) has been cited at least 194 times and validated for use in both western blotting and immunohistochemistry (https://www.thermofisher.com/antibody/product/Tau-Antibody-clone-HT7-Monoclonal/MN1000).

The mouse anti-phospho-tau 396/404 (PHF1) (Greenberg et al., J Biol Chem, 1992) (https://www.alzforum.org/antibodies/tau-phos-ser396ser404-phf-1) and mouse MC1 antibodies (Jicha et al., J Neurosci Res, 1997) (https://www.alzforum.org/antibodies/tau-mc1) were obtained from Dr. Peter Davies and have long been used and extensively validated in the field.

The rabbit anti-phospho-tau 199/202 antibody (Invitrogen, #44-768G) has been cited at least 35 times and validated for use in both western blotting and immunohistochemistry (https://www.thermofisher.com/antibody/product/Phospho-Tau-Ser199-Ser202-Antibody-Polyclonal/44-768G).

The rabbit anti-phospho-tau 356 antibody (Invitrogen, #44-751G) has been cited at least twice and validated for use in western blotting (https://www.thermofisher.com/antibody/product/Phospho-Tau-Ser356-Antibody-Polyclonal/44-751G). We also detected pS356 tau in western blotting at the expected molecular weight (Supplementary Fig. 7).

The mouse anti-beta-actin antibody (Cell Signaling Technology, #3700) has been cited at least 4170 times and extensively validated for use in western blotting (https://www.cellsignal.com/products/primary-antibodies/b-actin-8h10d10-mouse-mab/3700).

The rabbit anti-Iba1 antibody (FUJIFILM Wako, #0190-19741) has been commonly used to detect microglia, cited at least 4102 times, and extensively validated for use in immunohistochemistry (https://labchem-wako.fujifilm.com/us/product/detail/W01W0101-1974.html).

The rat anti-CD68 antibody (Bio-rad, #MCA1957) has been cited at least 224 times and validated for use in immunohistochemistry (https://www.bio-rad-antibodies.com/monoclonal/mouse-cd68-antibody-fa-11-mca1957.html?f=purified).

The rabbit anti-GFAP antibody (Abcam, #ab7260) has been commonly used to detect astrocytes, cited at least 1012 times, and validated for use in immunohistochemistry (https://www.abcam.com/products/primary-antibodies/gfap-antibody-ab7260.html).

For the rabbit anti-glucosylceramide (Glycobiotech, #RAS_0011) and rabbit anti-glucosylsphingosine antibodies (Antibody Research Corporation, #111584), we confirmed an increase in the immunoreactivity in the brains from GCase-inhibited mice in the present study.

The rabbit anti-GCase antibody (SIGMA, #G4171) has been cited at least 57 times and validated for use in western blotting and immunohistochemistry (https://www.citeab.com/antibodies/2296346-g4171-anti-glucocerebrosidase-c-terminal-antibody) (Qi et al.,Biochem J, 2019). We also observed correlation between the immunoreactive bands and GCase activities in the lysosomal-enriched fractions in the present study (Supplementary Fig. 15 and 16).

The mouse anti-phospho-alpha-synuclein antibody (81A) (BioLegend, #MMS-5091) has been cited at least 32 times and validated for use in immunohistochemistry (https://www.biolegend.com/fr-lu/products/purified-anti-alpha-synuclein-phospho-ser129-antibody-11221).

The rabbit anti-TDP-43 antibody (proteintech, #10782-2-AP) has been cited at least 1426 times and validated for use in immunohistochemistry (https://www.ptglab.com/products/TARDBP-Antibody-10782-2-AP.htm).

The rabbit anti-NeuN antibody (abcam, #ab177487) has been cited at least 695 times and validated for use in immunohistochemistry (https://www.abcam.com/products/primary-antibodies/neun-antibody-epr12763-neuronal-marker-ab177487.html).

The rabbit anti-FLAG antibody (SIGMA, #F7425) has been commonly used to detect FLAG-tagged proteins, cited at least 3120 times, and validated for western blotting (https://www.citeab.com/antibodies/1476271-f7425-anti-flag-r-antibody-produced-in-rabbit).

The mouse anti-mouse tau antibody, clone T49 (Not human) (SIGMA, #MABN827) has been cited at least 13 times and validated for use in immunostaining (https://www.citeab.com/antibodies/3289739-mabn827-anti-tau-antibody-clone-t49-not-human).

The rabbit anti-MAP2 antibody (Cell Signaling Technology, #4542) has been cited at least 164 times and validated for use in immunofluorescence (https://www.cellsignal.com/products/primary-antibodies/map2-antibody/4542).

The mouse anti-BMP antibody (Echelon, #Z-PLBPA) has been cited at least 48 times and validated for use in immunostaining (https://www.echelon-inc.com/product/purified-mouse-anti-lbpa-bmp/). It was used in immunostaining of a previous study (Logan et al., Cell 2021) and an decrease in the immunoreactivity was detected in PGRN-deficient samples, consistent with their lipidomics results.

The rabbit anti-LIMPII antibody (Novus Biologicals, #NB400-129) has been cited at least 24 times and validated for use in western blotting (https://www.novusbio.com/products/limpii-sr-b2-antibody_nb400-129).

The goat anti-mouse cathepsin B antibody (R&D, #AF965) has been cited at least 20 times and validated for use in western blotting (https://www.rndsystems.com/products/mouse-cathepsin-b-antibody_af965).

The goat anti-mouse cathepsin D antibody (R&D, #AF1029) has been cited at least 29 times and validated for use in western blotting (https://www.rndsystems.com/products/mouse-cathepsin-d-antibody_af1029).

The rabbit anti-Hsp60 antibody (Cell Signaling Technology, #4870) has been cited at least 123 times and validated for use in western blotting (https://www.cellsignal.com/products/primary-antibodies/hsp60-d307-antibody/4870).

The mouse anti-calreticulin antibody (Novus Biologicals #MAB38981) has been cited at least twice and validated for use in western blotting (https://www.novusbio.com/products/calreticulin-antibody-681233_mab38981). We also detected calreticulin in western blotting at expected molecular weight (Supplementary Fig. 14).

The rabbit anti-Rab5 antibody (Cell Signaling Technology #3547) has been cited at least 264 times and validated for use in western blotting (https://www.cellsignal.com/products/primary-antibodies/rab5-c8b1-rabbit-mab/3547).

The mouse anti-saposin C antibody (Santa Cruz #sc-347118) has been cited at lease twice and validated for use in western blotting (https://www.scbt.com/p/saposin-c-antibody-a-3). We also detected saposin C in western blotting at expected molecular weight (Supplementary Fig. 15 and 16).

Immunostaining results were also verified by omitting primary antibodies.

Eukaryotic cell lines

Policy information about cell lines and Sex and Gender in Research

Cell line source(s)	293T/17; Embryonic Kidney: Human (ATCC, CRL-11268)
Authentication	The cell line used was not authenticated.
Mycoplasma contamination	The cell line used tested negative for mycoplasma contamination.
Commonly misidentified lines (See ICLAC register)	No commonly misidentified cell lines were used in this study.

Animals and other research organisms

Policy information about studies involving animals; ARRIVE guidelines recommended for reporting animal research, and Sex and Gender in Research

Laboratory animals	Human P301S tau transgenic (PS19) mice with B6C3F1 background were obtained from the Jackson Laboratory (JAX:008169). Grn−/− mice with C57BL/6J background were obtained from the RIKEN Bioresource Center (RBRC02370). To generate 6 genotypes used in this study (WT, Grn+/−, Grn−/−, PS19, PS19 Grn+/−, and PS19 Grn−/−) with minimal differences in genetic background, PS19 and Grn −/− mice were first crossed to generate PS19 Grn+/− and Grn+/− mice and then these mice were crossed. The resulting littermates with WT, Grn+/−, Grn−/−, PS19, PS19 Grn+/−, or PS19 Grn−/− genotype born with the ratio 1:2:1:1:2:1 were used in the experiments. All behavioral tests were performed at 10-11 months of age. Volumetric, Immunohistochemical, and biochemical analyses were performed at 9-12 months of age (mean ages are not significantly different between genotypes in all analyses).
Wild animals	This study did not involve wild animals.
Reporting on sex	For behavioral tests, both male and female mice were used with similar ratio between genotypes. Data using only male mice are

	provided in Supplementary Fig. 1. For volumetric, immunohistochemcal, and biochemical analyses, only male were used as previous studies have reported significantly more tau pathology and neurodegeneration in male versus female PS19 mice (Leyns et al., 2017; Shi et al., 2017; Wu et al., 2019; Yanamandra et al., 2013)
Field-collected samples	This study did not involve samples collected from the field.
Ethics oversight	All protocols were approved by Yale Institutional Animal Care and Use Committee (IACUC) (2023-07281).

Note that full information on the approval of the study protocol must also be provided in the manuscript.

Plants

Seed stocks	n/a
Novel plant genotypes	n/a
Authentication	n/a
